# Site-Specific Gene Knock-Out and On-Site Heterologous Gene Overexpression in *Chlamydomonas reinhardtii* via a CRISPR-Cas9-Mediated Knock-in Method

**DOI:** 10.3389/fpls.2020.00306

**Published:** 2020-03-20

**Authors:** Jongrae Kim, Sangmuk Lee, Kwangryul Baek, EonSeon Jin

**Affiliations:** Department of Life Science, Research Institute for Natural Science, Hanyang University, Seoul, South Korea

**Keywords:** *Chlamydomonas reinhardtii*, genetic modification, CRISPR-Cas9, knock-in, on-site gene overexpression

## Abstract

*Chlamydomonas reinhardtii* is being transformed from a model organism to an industrial organism for the production of pigments, fatty acids, and pharmaceuticals. Genetic modification has been used to increase the economic value of *C. reinhardtii*. However, low gene-editing efficiency and position-effects hinder the genetic improvement of this microorganism. Recently, site-specific double-stranded DNA cleavage using CRISPR-Cas9 system has been applied to regulate a metabolic pathway in *C. reinhardtii*. In this study, we proved that site-specific gene expression can be induced by CRISPR-Cas9-mediated double-strand cleavage and non-homologous end joining (NHEJ) mechanism. The CRISPR-Cas9-mediated knock-in method was adopted to improve gene-editing efficiency and express the reporter gene on the intended site. Knock-in was performed using a combination of ribonucleoprotein (RNP) complex and DNA fragment (antibiotics resistance gene). Gene-editing efficiency was improved via optimization of a component of RNP complex. We found that when the gene *CrFTSY* was targeted, the efficiency of obtaining the desired mutant by the knock-in method combined with antibiotic resistance was nearly 37%; 2.5 times higher than the previous reports. Additionally, insertion of a long DNA fragment (3.2 and 6.4 kb) and site-specific gene expression were analyzed. We demonstrated the knock-out phenotype of *CrFTSY* and on-site inserted gene expression of luciferase and mVenus at the same time. This result showed that CRISPR-Cas9-mediated knock-in can be used to express the gene of interest avoiding position-effects in *C. reinhardtii*. This report could provide a new perspective to the use of gene-editing. Furthermore, the technical improvements in genetic modification may accelerate the commercialization of *C. reinhardtii*.

## Introduction

*Chlamydomonas reinhardtii* is widely used as a model organism and considered to be a potential cell factory to produce value-added compounds (Khan et al., 2018; Salomé and Merchant, 2019). Production of compounds such as zeaxanthin, sesquiterpene, bio-hydrogen, and human epidermal growth factor, have been reported in *C. reinhardtii* (Torzillo and Seibert, 2013; Lauersen et al., 2016; Baek et al., 2018; Baier et al., 2018a). These reports have increased the attention on the commercial use of *C. reinhardtii*. Moreover, the availability of *C. reinhardtii* as a biotechnological platform has been maximized through easy genetic modification techniques (Scaife et al., 2015).

Genetic modifications have been used to enhance the production of value-added compounds and produce fine chemicals in *C. reinhardtii* (Fu et al., 2019; Salomé and Merchant, 2019). Although gene transformation in *C. reinhardtii* is well-developed and easy, gene overexpression in *C. reinhardtii* is considered to be a huge obstacle for the advancement of the industry (Doron et al., 2016). Strategies such as developing a strong promoter (Kumar et al., 2013; Scranton et al., 2016; López-Paz et al., 2017), increasing translation using introns (Baier et al., 2018b), increasing gene expression stability using self-cleaving peptides (Rasala et al., 2012; Plucinak et al., 2015), developing a new reporter system using ferredoxin fused hydrogenase for effective screening (Eilenberg et al., 2016), and sequence-based optimization of RNA transcription (Weiner et al., 2018) have been developed for enhancing gene overexpression. The gene expression system is gradually being optimized by improving the technique. Though gene expression is notably advanced in *C. reinhardtii*, random insertion of the transformed gene is still problematic (Weiner et al., 2018; Jia et al., 2019). The random insertion leads to different levels of protein expression of the same gene called position-effect and also causes unexpected mutations. Therefore, the present study aimed to improve the heterologous gene expression technique by avoiding position-effects and inserting genes effectively at the desired site.

For the specific gene knock-out, gene-editing techniques like zinc-finger nuclease (ZFN) and transcription activator-like effector nuclease (TALEN) are used to create specific double-stranded DNA cleavages (Gaj et al., 2013). However, these techniques have high off-target mutation tendency and low feasibility (Gupta and Musunuru, 2014). Using the clustered regularly interspaced short palindromic repeats-associated protein 9 (CRISPR-Cas9) in eukaryotes, researchers can edit (cleave and knock-out) specific locations on genome more easily (Jinek et al., 2012; Cho et al., 2013; Cong et al., 2013; Mali et al., 2013). CRISPR-Cas9 system requires three basic components: the nuclease CRISPR-associated protein 9 (Cas9) cleaving the *RuvC* site which is 3-nucleotide far from PAM, a CRISPR RNA (crRNA) containing a 20-base pair sequence complementary to the target DNA, and a trans-activating crRNA (tracrRNA). crRNA and tracrRNA can be physically linked to form single guide RNA (gRNA) (Jinek et al., 2012). In *C. reinhardtii*, the CRISPR-Cas9 was first applied via DNA vector system in 2014 and recently ribonucleoprotein (RNP) system has been developed (Jiang et al., 2014; Baek et al., 2016; Guzmán-Zapata et al., 2019). CRISPR-Cas9 system is the ideal tool for gene-editing; however, it requires efficient selective markers for reducing the time and labor. Presently, phenotypic changes such as visual changes are used to improve the selection efficiency, however, the method is less efficient, requires a lot of labor (Baek et al., 2016; Shin et al., 2016; Greiner et al., 2017; Jeong et al., 2017b), and is not suitable for most genes. Therefore, gene-editing methods using counter-selective markers such as adenine phosphoribosyl transferase (APT), nitrate reductase (NR), peptidylprolyl isomerase (FKB12), tryptophan synthase beta subunit (TSB), and orotidine 5′-phosphate decarboxylase (UMP) have been recently proposed (Shin et al., 2016; Wang et al., 2016; Jiang and Weeks, 2017; Serif et al., 2018; Guzmán-Zapata et al., 2019). Counter selection can be efficient; however, they are eventually dependent on specific phenotypes. To overcome this limitation, pre-selection using antibiotic resistance for enhancing the efficiency of gene-editing has been reported (Shin et al., 2016; Jiang and Weeks, 2017). However, the conditions have not been optimized and the gene-editing efficiency reported has not exceeded >15% till date (Greiner et al., 2017).

As mentioned above, advanced gene expression tools and gene-editing techniques have played a major role in increasing the commercial use of *C. reinhardtii*. However, the efficiency of these methods is low and the rate of unintended mutations is high. In this study, to develop the new technique of genetic modification in *C. reinhardtii*, we investigated the knock-in method to improve gene-editing efficiency while inducing gene expression at the desired location. Phenotypic studies indicated that the two desired characteristics were obtained simultaneously. We optimized the technique that increased gene-editing efficiency to >30% and successfully demonstrated site-specific gene expression by avoiding random mutations.

## Materials and Methods

### Culture Condition

*Chlamydomonas reinhardtii* CC4349, CC124, and CC503 (purchased from *Chlamydomonas* Resource Center, University of Minnesota, United States) were maintained photoheterotrophically in Tris-Acetate-Phosphate (TAP) medium at 25°C with continuous light (80 μmol photons m^–2^ s^–1^) on an orbital shaker (100 rpm). The cells were cultivated till the log phase in liquid TAP medium under the same conditions for all the experiments. For the selection and maintenance of transformant lines, solid TAP medium fortified with 30 μg/mL hygromycin-B (Thermo Fisher, MA, United States) for ΔCrFTSY-Ga and with 30 μg/mL hygromycin-B, 30 μg/mL paromomycin (Sigma-Aldrich, MO, United States) for ΔCrFTSY-mV, were used.

### Knock-in Mutant Generation by CRISPR-Cas9

**FIGURE 1:**
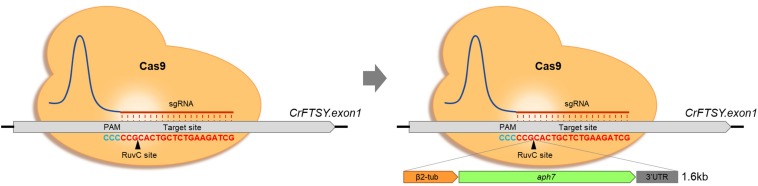
Optimization of gene-editing condition based on CRISPR-Cas9-mediated knock-in strategy. Formal knock-out method by CRISPR-Cas9 **(Left)** and knock-in with donor DNA **(Right)**. The inserted DNA fragment consisted of β2-tubulin promoter, aph7 (hygromycin-B resistance), rbcs2 3′UTR.

**FIGURE 2:**
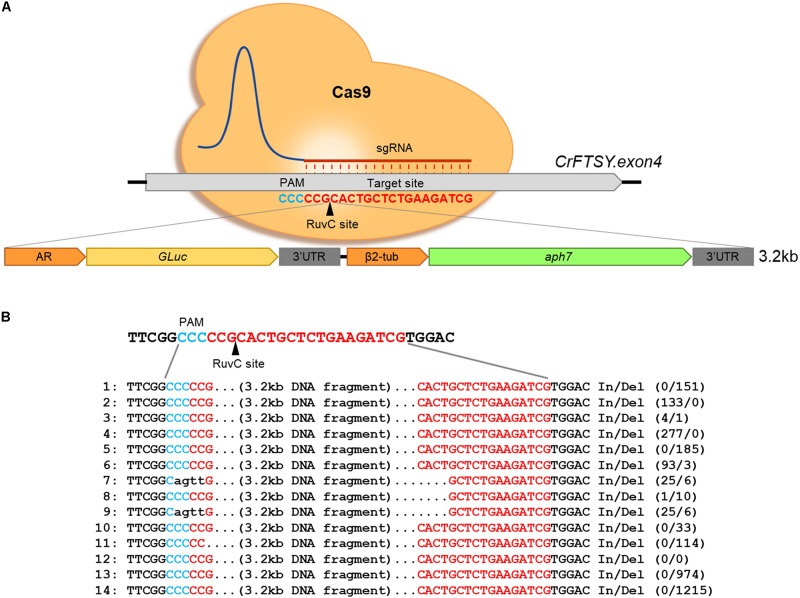
Target-specific long length DNA insertion via CRISPR-Cas9-mediated knock-in method. **(A)** Schematic diagram of large DNA fragment insertion in *CrFTSY*. Target-specific DNA cleavage via CRISPR-Cas9 and insertion of the large gene of interest (3.2 kb, *GLuc*-*aph7* DNA cassette) simultaneously. **(B)** Sequence analysis done by the Sanger sequencing method to identify the inserted DNA fragment on Δ*CrFTSY*-Ga.

To optimize the gene-specific knock-out efficiency, RNP method was used and the optimal conditions were determined with slight modification according to the method described previously (Baek et al., 2016). The target sequence of *CrFTSY* and the gRNA sequence, 5′-CGATCTTCAGAGCAGTGCGG-3′, that was the same as that of the previous study (Baek et al., 2016), were used to avoid the off-target effect. Cas9 protein was purchased from ToolGen Inc. (Seoul, South Korea) and gRNA was synthesized *in vitro* using GeneArt^TM^ Precision gRNA Synthesis Kit (Thermo Fisher, MA, United States) following the manufacturer’s protocol. The inserted DNA fragments, *aph7* gene (aminoglycoside phosphotransferase7, resistance against hygromycin B) (1.6 kb) (Figure 1 left) and *aph7-GLuc* (Gaussia Luciferase) (3.2 kb) (Figure 2A) DNA cassettes, were amplified by polymerase chain reaction using amplifying primers (sense: 5′-CGGTTCCTGGCCTTTTGCTGG-3′ and antisense: 5′-CAAGTACCATCAACTGACGTTACATTCTG-3′). The inserted DNA fragment, *aph8* (aminoglycoside phosphotransferase8, resistance against paromomycin) – *mVenus* (yellow fluorescence protein) – *aph7* (6.4 kb) (Figure 6A) DNA cassette, was linearized by restriction enzyme (*Sca*I, *Spe*I). All the inserted DNA fragments were purified by agarose gel extraction.

To generate the target-specific knock-in mutant using RNP in *Chlamydomonas*, 2 × 10^7^ cells were transformed with Cas9 protein premixed with gRNA (RNP complex). Lyophilized Cas9 protein was dissolved in gRNA solution prepared with nuclease-free water and the mixture was incubated for 10 min at room temperature (20–25°C) to make a complex. Cells mixed with the RNP complex were transferred into 4 mm cuvettes and transformed by Gene Pulser Xcell^TM^ Electroporation Systems (Bio-Rad, CA, United States) set at 600 V, 50 μF, and 200 Ω. After transformation, the cells were incubated overnight (16–20 h) and spread on the selective medium. Colonies appeared within 2 weeks. The antibiotic resistance colonies were transferred to liquid medium for the sequence analysis. This pre-selected colony number is classified as “candidate” for the calculation of knock-in efficiency.

### Identification of DNA Sequence of CrFTSY and Inserted DNA Sequence

The genomic DNA was prepared from the colonies grown on selective medium using the Chelex-100 method (Wan et al., 2011). Targeted region (around 200 bp from gRNA sequence) was amplified by PCR with specific primers (sense: 5′-GGTGTCCCCGCAATCACCAAC-3′, antisense: 5′-CACCCACACCCACCTTGAGCGAG-3′). The PCR product was purified by agarose gel electrophoresis and sequenced using Sanger sequencing by Macrogen Inc. (Seoul, South Korea).

### Measurement of Chlorophyll Content

Wild-type and mutant cells were cultured in liquid TAP medium untill the mid-log phase. To make comparison of colorimetric analysis, the cultured cells were collected from the liquid media, and 2.5 × 10^6^ cells were deposited on solid TAP medium. The cells were incubated for 24 h and the difference in color characteristics was compared. For measurement of the chlorophyll contents, 1 × 10^7^ cells were suspended in 80% (v/v) acetone, and the absorbance was measured by spectrophotometer at wavelength of 663 and 647 nm. The formula used for calculating the content is given by Melis et al. (1987).

### Measurement of Gaussia Luciferase Activity

Luciferase activity was measured by a previously reported protocol (Kim et al., 2018) with slight modifications to confirm *GLuc* expression. *GLuc* is secreted in the medium hence, its activity was measured using the complete cell culture (Ruecker et al., 2008) and it is more sensitive than using only the cells. The mutant cells were cultured till the absorbance at 750 nm reached 1.0 indicating mid-log phase. A small volume (200 μL) of culture was used for the luciferase assay using the *Renilla* Luciferase Assay Kit (Promega, WI, United States). The cells were suspended in 40 μL of lysis buffer and lysed by vigorous vortexing. The lysed cells were pelleted by centrifugation at 13,000 rpm for 5 min. Then, 80 μL supernatant was mixed with equal volume of substrate solution. The chemiluminescence was measured immediately after mixing the two solutions in a Glo Max^TM^ 20/20 luminometer (Promega, WI, United States). The statistical difference was analyzed by Student’s *t*-test (*p*-value < 0.05).

### Fluorescence Microscopic Analysis

ΔCrFTSY-mV was cultivated in selective medium as mentioned above. Five microliters of cultured cells were dropped on a glass slide and covered with a coverslip. Fluorescence was detected by a fluorescence microscope (Eclipse Ni, Nikon, Tokyo, Japan). Fluorescence detection wavelengths were 540 ± 20 nm with YFP filter for mVenus and 630 ± 30 nm with Texas RED filter for auto-fluorescence of chlorophyll. The magnification was 400×.

### Southern Blot Analysis

Southern blot was used to confirm single DNA insertion in mutants. Genomic DNA was extracted from cells in the log phase by the CTAB method (Porebski et al., 1997). The genomic DNA was isolated (20 μg) and digested by *Pvu*II or *Sma*I restriction enzymes. Digested genomic DNA was separated using 0.8% agarose gel and transferred to a nylon membrane by capillary transfer. Membrane attached DNA was developed through hybridization with an aph7 probe and amplified by PCR with specific primers (sense: 5′-ATGATTCCTACGCGAGCCTG-3′, antisense: 5′-ATCCGGCTCATCACCAGGTA-3′) using the Gene Images AlkPhos Direct Labeling and Detection System (Amersham, Little Chalfont, Bucks, United Kingdom). The process was carried out according to the manufacturer’s protocol.

### Western Blot Analysis

Cultivated cells (1 × 10^6^) were boiled in SDS-PAGE loading buffer, electrophoresed on 15% SDS-polyacrylamide gels, and transferred to a PVDF membrane using Xcell II blot module (Thermo Fisher, MA, United States). GLuc antibody (NEB, MA, United States) was used to detect GLuc protein. ATP-β antibody (Agrisera, Vännäs, Sweden) was used as a reference. HRP-conjugated goat anti-rabbit IgG (H + L) antibody (Life Technologies, CA, United States) was used as a secondary antibody. GLuc and ATP-β were visualized on an X-ray film by chemiluminescence using EPD Western Reagent (ELPIS-BIOTECH, Daejeon, South Korea).

## Results

### Improvement of Gene-Editing Efficiency by CRISPR-Cas9-Mediated Knock-in

In this study, the selective marker system *aph7* DNA cassette was used to optimize the knock-in method in *C. reinhardtii*. This strategic knock-in method was induced by CRISPR-Cas9-mediated knock-out, and the selective marker gene was inserted into the cleaved site by NHEJ (Figure 1). The mutants were screened more effectively by the expression of the antibiotic selective marker in the inserted DNA fragment.

Although the knock-in method has been reported previously, the gene-editing efficiency was very low (under 15%). Therefore, in this study gene-editing efficiency was analyzed by using different concentrations of RNP complex required for transformation (Table 1). Every single experiment resulted in a different number of colonies but a similar percentage of positive colonies (Supplementary Figure 1). The gene-editing efficiency of *CrFTSY* (Δ*CrFTSY*) was observed to be maximum at 36.8%, when transformed with 100 μg Cas9 and 70 μg gRNA. Even on transformation with 10 μg Cas9 and 7 μg gRNA, Δ*CrFTSY* efficiency was >16.5%, which was higher than previously reported (Baek et al., 2016; Shin et al., 2016; Greiner et al., 2017). Thus, we optimized the concentration of RNP complex for maximum gene-editing efficiency. Additionally, we investigated the gene-editing efficiency depending on the presence or absence of a cell wall. The gene-editing efficiency in *C. reinhardtii* strains CC503 (no cell wall) and CC124 (with cell wall) were observed under the same method and it was found to be similar to that of *C. reinhardtii* CC4349 (Supplementary Figure 2). This result indicated that the knock-in method is universally applicable independent of the cell type.

**TABLE 1:** Gene-editing efficiency obtained via the knock-in method.

	**RNP complex**							
Cas9 protein (μg)	200	10	1.6	200	100	50	20	10
gRNA (μg)	140	7.5	0.32	140	70	35	14	7
Donor DNA (μg)	0	1	0.3	1	1	1	1	1

	**Gene-editing efficiency**								

Positives (%)	0.56	1.4	14.8	35.2	36.8	33.8	27.4	16.5
References	Baek et al., 2016	Shin et al., 2016	Greiner et al., 2017	In this study				

### Insertion of Long-Length DNA Fragment on Intended Site

In the gene expression using foreign DNA, transformed foreign DNA integrates in the nuclear genome of *C. reinhardtii*. This DNA integration in the genome predominantly leads to unexpected mutations and position-effects (Leon and Fernandez, 2007). Therefore, we investigated the possibility of on-site foreign gene expression through the knock-in method to reduce position effects. Firstly, a 3.2 kb long DNA fragment was transformed into the target site of by the knock-in method described above (Figure 2A). Large DNA insertion in the target site was confirmed by genomic PCR in 14 positives among 39 candidates (36% gene-editing efficiency) of ΔCrFTSY_Ga colonies in which *GLuc*-*aph7* DNA cassette was inserted into *CrFTSY* (Supplementary Figure 3). ΔCrFTSY-Ga DNA was sequenced and compared with the expected sequence after integration (Figure 2B and Supplementary Data Sheet 2). Targeted DNA sequences on *CrFTSY* were neatly cleaved by Cas9 in ΔCrFTSY-Ga mutants except mutants 7 and 9. Among the 14 positive mutants, clean insertion without any In/Del was detected only in mutant 12. As evident in this result, the inserted DNA sequence resulted in mutations during integration into the genome. Thus, it is important to identify the exact sequence of the insertion site. Although the problems related to the mutations of inserted DNA remain unsolved, however, this result showed that the insertion of DNA longer than 3 kb is possible at the desired site.

### Expression of the Foreign Gene at the Desired Site by Knock-in

**FIGURE 3:**
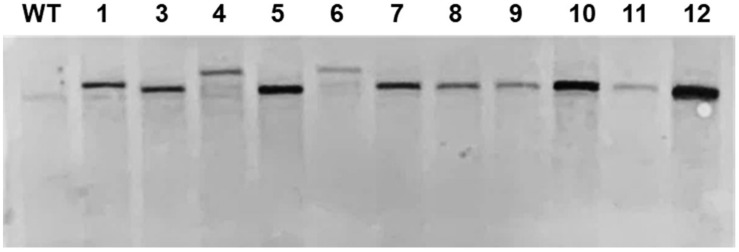
Southern blot for validation of copy number of an inserted DNA fragment. Extracted genomic DNA was digested by *Sma*I and blotted on a nylon membrane. The *aph7* probe was used for detection of DNA integration.

We also analyzed the expression of the foreign genes at the desired site. To confirm the normal expression of *GLuc* inserted at the target site, we confirmed the copy number of DNA insert in ΔCrFTSY-Ga mutants. Mutant 2 was excluded in the further analysis as it was mixed with non-mutant cells. Mutants 13 and 14 were also excluded as the promoter sequence of *GLuc* got deleted. Southern blot analysis was performed to determine the copy number of DNA insert. Genomic DNA was digested by restriction enzymes and hybridized with the specific probe. All ΔCrFTSY-Ga mutants had a single copy of the DNA insert (Figure 3 and Supplementary Figure 4). This result suggests that luciferase activity observed in the next experiment was due to a single Gaussia luciferase gene inserted on *CrFTSY*.

**FIGURE 4:**
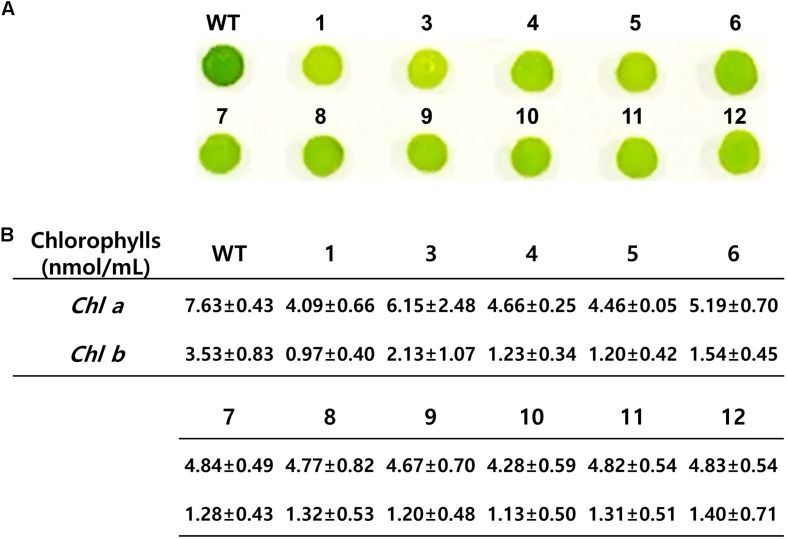
Phenotypic characteristics of *CrFTSY* knock-out. Wild-type and ΔCrFTSY-Ga mutants grew on TAP agar medium under 80 μmol photon m^–2^ s^–1^ light. **(A)** Pale green color was detected in ΔCrFTSY-Ga mutants compared to dark green color exhibited by wild type. **(B)** Amount (nmol mL^–1^) of Chlorophyll-a and -b of ΔCrFTSY-Ga mutants (*n* = 3; the values shown are means ± SD).

*Chlamydomonas reinhardtii* with mutation in *CrFTSY* appeared to be pale green in color compared to the color of the wild type due to the reduction in chlorophyll content on a theoretical basis (Kirst et al., 2012). We observed that 11 ΔCrFTSY-Ga mutants obtained were pale green in color compared to that of the wild type on solid TAP medium (Figure 4A). Moreover, to validate this visual difference between WT and mutants, we measured the chlorophyll content of all the samples, including WT (Figure 4B). The levels of chlorophyll-a (4.80 ± 0.76 nmol mL^–1^) and -b (1.34 ± 0.53 nmol mL^–1^) in ΔCrFTSY-Ga mutants were reduced to 63 and 38% of wild type chlorophyll-a and chlorophyll-b (7.63 ± 0.43 and 3.53 ± 0.83), respectively. Therefore, the chlorophyll a/b ratio was increased by 1.8 ± 0.2-fold in ΔCrFTSY-Ga mutants compared to that of wild type, as also shown in the previous report (Baek et al., 2016). The results clearly reflected the phenotypic differences when the *FTSY* was knocked out (Figure 4).

**FIGURE 5:**
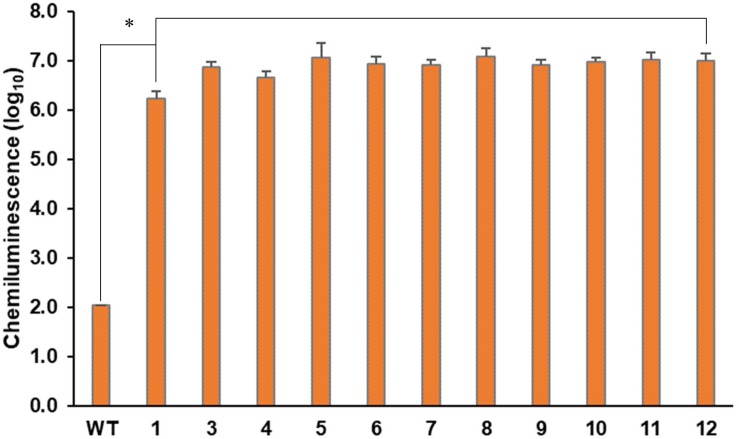
Phenotypic characteristics of expression of the inserted DNA fragment. *Gaussia* luciferase assays from ΔCrFTSY-Ga mutants were performed in triplicate independently. The asterisk indicates the statistical difference determined by Student’s *t*-test (*n* = 3; the values shown are means ± SD; *p* < 0.05).

The foreign gene expression on the intended site was verified by confirming the protein expression of GLuc (Supplementary Figure 5) and measuring the luciferase activity (Figure 5). We used the cultured cells and medium together for the luciferase analysis as mentioned before. As shown in Figure 5, all Δ*CrFTSY*-Ga mutants successfully expressed *GLuc* while luciferase activity was negligible in the wild type. The results of this experiment demonstrated that a gene of interest can be expressed on the desired site without random insertional mutation. Mean difference between *GLuc* activity in 11 different mutants was 20%, which was significantly lower than the mean difference (75%) found in random integration mutants (Kim et al., 2018). This constant expression level in 11 mutants was possible due to the insertion of a single copy of DNA fragment at the intended site on the genome.

### Determination of the Maximum Size of DNA Fragment Inserted on Target by Knock-in

**FIGURE 6:**
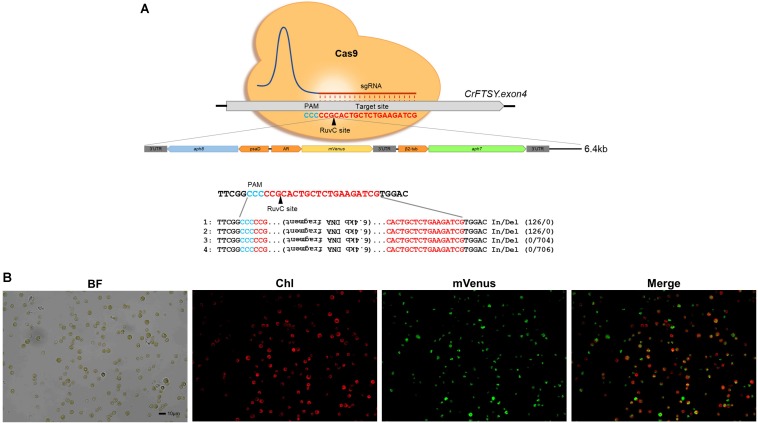
Target specific 6.4 kb long DNA (*aph8*–*mVenus*–*aph7* DNA cassette) insertion via CRISPR-Cas9-mediated knock-in. **(A)** Sequence analysis of target site and inserted DNA fragment. **(B)** Visualization of mVenus expression in Δ*CrFTSY*_mV at insertion site by fluorescence microscopy (red, chlorophyll; green, *mVenus*). Scale bar indicates 10 μm.

We tested the possibility of insertion of a DNA fragment larger than 3.2 kb through the knock-in method, which confirmed the insertion of a 6.4 kb long DNA fragment in the target site. For this additional experiment, we used the same methodology as described in the section “Expression of the Foreign Gene at the Desired Site by Knock-in.” The DNA insertion in the target site was confirmed by genomic PCR. Four positives among 12 candidates (33% gene-editing efficiency) of ΔCrFTSY_mV in which *aph8*-*mVenus*-*aph7* DNA cassette was inserted into *CrFTSY* were obtained (Supplementary Figure 6). From the sequence analysis at the insertion site, we found long length In/Del mutations (Figure 6A and Supplementary Data Sheet 2). Two mutants (mutants 1 and 2) had 126 bp insertion and two different mutants (mutants 3 and 4) had around 700 bp deletion. As in the above result (Figure 2), In/Del occurred non-specifically in this experiment. Nevertheless, the knock-in method deleted the desired genes and reconfirmed that the introduction of DNA fragment for overexpression was effective. mVenus expression in the selected mutant was visualized by fluorescence microscopy (Figure 6B). Hence, we confirmed that a DNA fragment of up to 6.4 kb long could be inserted at the desired location and overexpressed. From these results, we demonstrated that the CRISPR-Cas9-mediated knock-in method was an effective method that allowed the gene deletion and overexpression of foreign genes in a single experiment.

## Discussion

Increasing the commercial use of *C. reinhardtii* requires improvement of the strain by genetic modification for the production of high-value compounds. *C. reinhardtii* has been extensively studied for fundamental research and industrial use based on its genome sequence data and well-developed molecular tool kit (Scaife et al., 2015; Crozet et al., 2018; Salomé and Merchant, 2019). Moreover, the genetic modification techniques are highly developed and the engineering strategies of metabolic pathways are well established (Plucinak et al., 2015; Baier et al., 2018b; Fu et al., 2019; Kong et al., 2019).

Despite several refined concepts, the improvement of *C. reinhardtii* is still not sufficient and simple. Due to the non-specific disruption of genes generating the desired mutants has been difficult, hampering reverse-genetic studies (Fu et al., 2019; Park et al., 2019). The existing transformation methods of *C. reinhardtii* cannot target a specific gene, thus researchers cannot regulate precisely the desired genes (Leon and Fernandez, 2007; Jia et al., 2019; Kim et al., 2019). These problems can be overcome by the recently developed gene-editing techniques. Gene-editing techniques based on RNP using Cas9 proteins are being recognized as the most effective gene specific knock-out methods to date (Patel et al., 2019). Cas9-mediated gene knock-out has been reported for several genes and the use of donor DNA with RNP, called knock-in, has emerged recently. However, the gene-editing efficiency reported in previous studies was not satisfactory (Shin et al., 2016; Greiner et al., 2017; Jeong et al., 2017a).

Recently, some reports suggested that gene selection can be achieved with high yields (up to 30%) through counter selection without the use of antibiotic genes (Jiang and Weeks, 2017; Serif et al., 2018; Guzmán-Zapata et al., 2019). However, these methods are mostly functional for specific genes and cannot be applied universally.

In this study, we used the antibiotic gene (*aph7*) as donor DNA to ensure high selection efficiency for the optimization of the Cas9-mediated knock-in method. In contrast to the previously reported CRISPR-Cas9-mediated knock-out methods, the use of a selective marker in the knock-in method of our study enhanced the knock-out efficiency by inserting of an external DNA into the cleaved site (Figure 1). The method of using the antibiotic gene employed in this study has been proven to be generally applicable while effectively performing the gene-editing of other genes (*AGP* and *LCYE*) (unpublished data). Therefore, we suggest that the use of antibiotic genes as donor DNA is an efficient method when using the RNP-based gene-editing. In addition, through the optimization of RNP complex used in this study, the gene-editing efficiency was increased up to 37% (Table 1 and Supplementary Figure 1). These results indicate that the RNP complex of 100 μg:70 μg (Protein:gRNA) is optimal for the target specific gene editing in *C. reinhardtii*.

An additional benefit of establishing the knock-in method is that it avoids the position-effects of random mutations that occur during the transformation process. We strategically utilized this methodology to validate the expression of the gene of interest at the desired position (Figures 2–5). The successful expression of the report gene (*GLuc* and *mVenus*) and the fact that the single copy of DNA insert was integrated into genome showed that our strategy of avoiding position-effect and expressing the gene of interest at the target location successful (Figures 3–6). The success of our target-oriented gene insertion strategy could provide a new strategic perspective for future *C. reinhardtii* improvement studies.

In this paper, we reported an improved gene-editing technique. Despite several improvements in different techniques, researchers still face the problem of tedious processes for species improvement. Even though the target-oriented gene insertion based on knock-in method was performed, validation for the precise knock-in was still required. Besides, due to the lack of information on the sites effectively targeted by Cas9, the location of gene insertion is limiting, which unavoidably involves the deletion of one gene. Therefore, it is important to discover target locations with high gene-editing efficiency without affecting the biological function of the cell. As our results show in Figures 2, 6, the inserted gene was integrated into genomic DNA by NHEJ, therefore it is necessary to develop a technique to prevent the mutation in sequences in the integration process.

## Data Availability Statement

The raw data supporting the conclusions of this article will be made available by the authors, without undue reservation, to any qualified researcher.

## Author Contributions

EJ and JK conceived and designed the experiments. KB confirmed the optimal condition of RNP. JK and SL performed the knock-in study and analyzed the gene expression. JK, SL, and EJ wrote the manuscript. All authors reviewed the manuscript.

## Conflict of Interest

The authors declare that the research was conducted in the absence of any commercial or financial relationships that could be construed as a potential conflict of interest.
