# Supplementary material for: Site-Specific Gene Knock-Out and On-Site Heterologous Gene Overexpression in Chlamydomonas reinhardtii via a CRISPR-Cas9-Mediated Knock-in Method
Source: Front Plant Sci. 2020 Mar 20;11:306. doi: 10.3389/fpls.2020.00306 (PMC7099044; doi:10.3389/fpls.2020.00306)
Supplement: Supplementary file 1 [file Data_Sheet_1.docx]

Supplementary Material


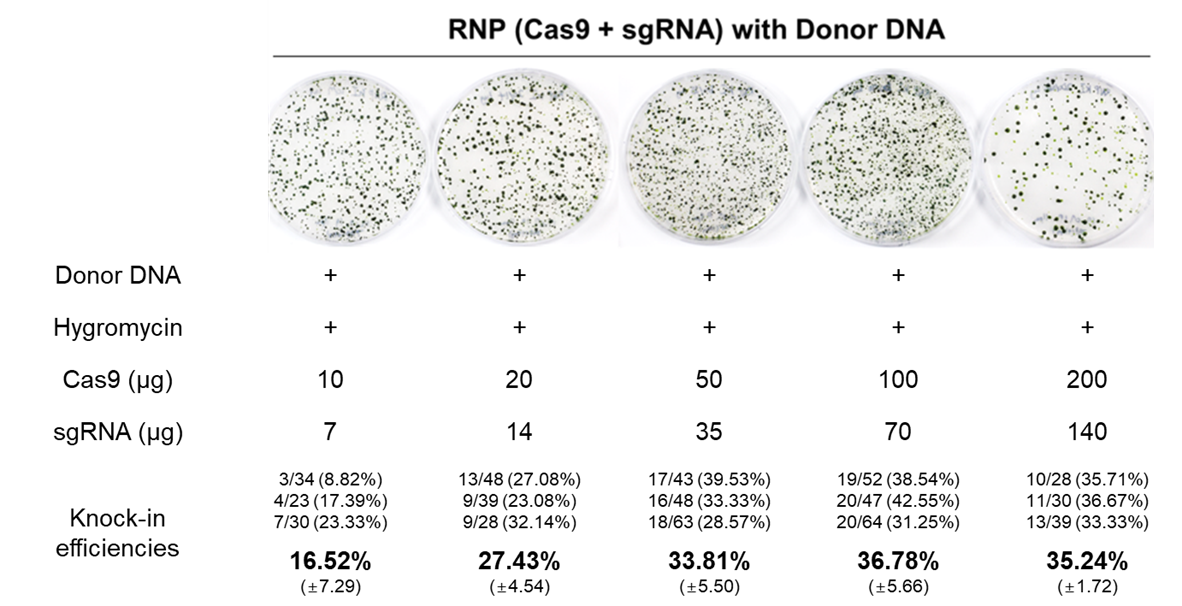


**Supplementary Figure 1**. Improvement of gene-editing efficiency based on CRISPR-Cas9 using additional selection strategy with donor DNA. Gene-editing efficiency increased with gradual increase in Cas9 and gRNA amounts from (10 + 7) µg to (100 + 70)µg Cas9+gRNA.


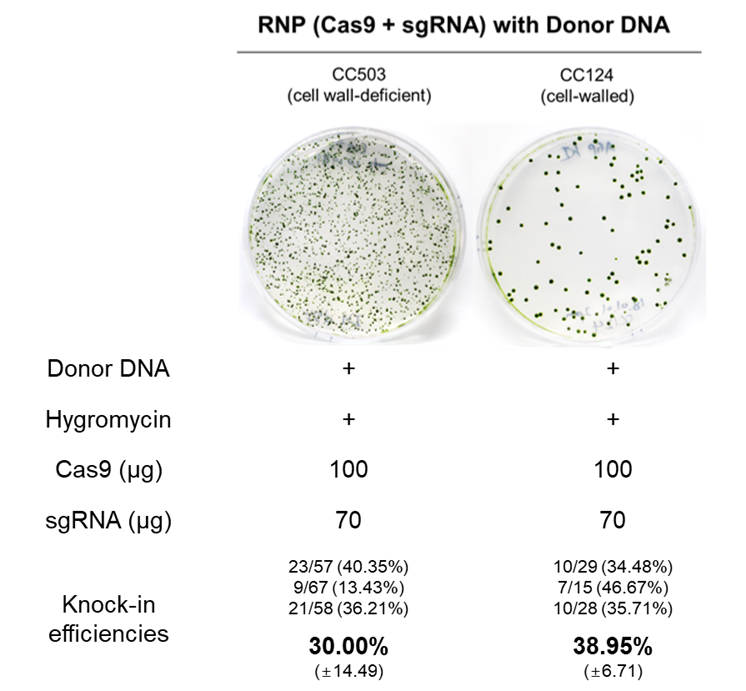


**Supplementary Figure 2**. Validation of gene-editing efficiency in different cell types. Mutation efficiency of *C. reinhardtii* CC503 (cell wall-deficient) and CC124 (cell-walled) strains.


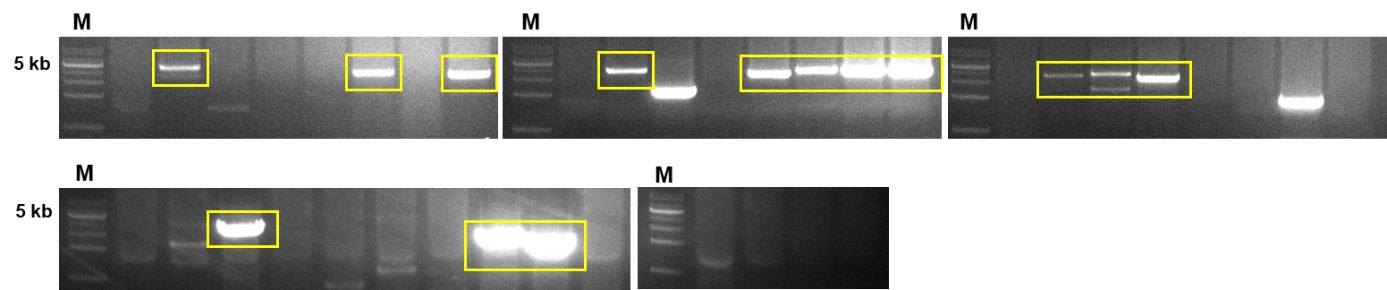


**Supplementary Figure 3**. Target-specific DNA insertion (3.2 kb) conjugated with CRISPR-Cas9. PCR of DNA extracted from pale green colonies of *CrFTSY* knock-out *C*. *reinhardtii*. PCR product bigger than 3 kb (marked by the yellow box) is detected only in the candidates where DNA fragment was inserted. 14 positive colonies of 39 candidates were confirmed. M, 1 kb DNA ladder.

(a)


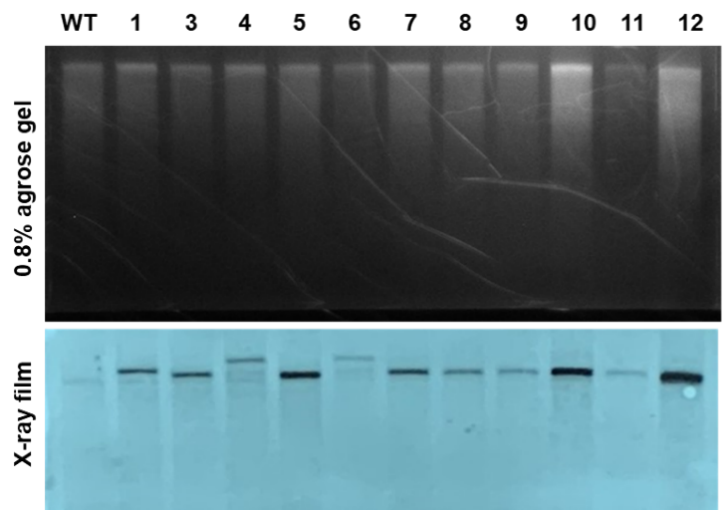


(b)


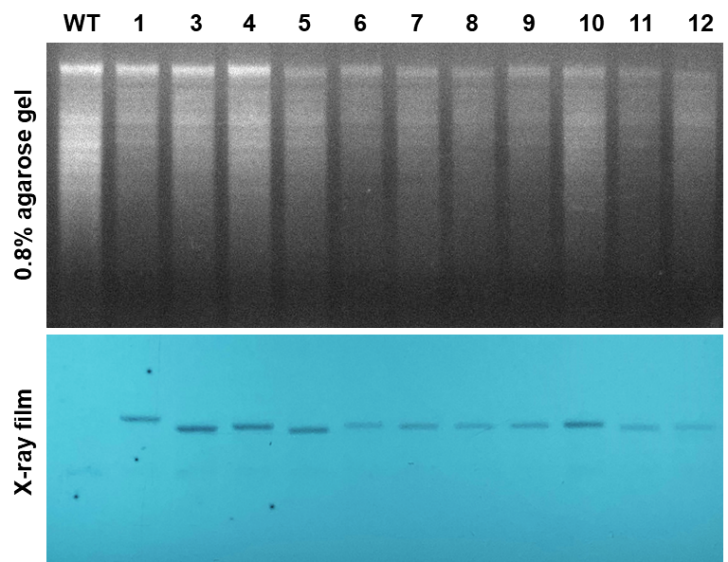


**Supplementary Figure 4**. Southern blot for validation of copy number of inserted DNA fragment. Extracted genomic DNA was digested by a) *Sma I* and b*) Pvu II* blotted on membrane. *aph7* probe was used for detection of DNA integration. Luminescence signal was visualized on X-ray film.

(a)


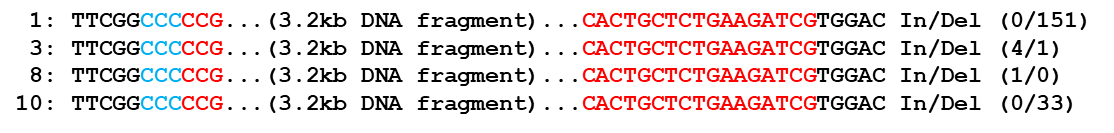


(b)


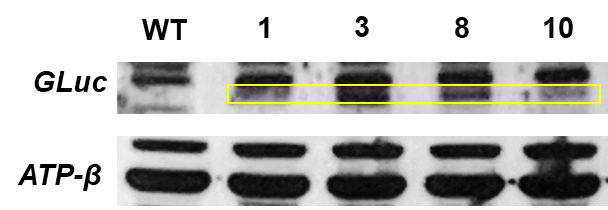


**Supplementary Figure 5**. Western blot for validation of heterologous gene expression. a) Four different transformants were chose for the analysis based on their sequence on In/Del, b) Expression of *Gaussia* luciferase was detected by specific antibody (upper layer). *ATP-β* antibody was used as reference (down layer).


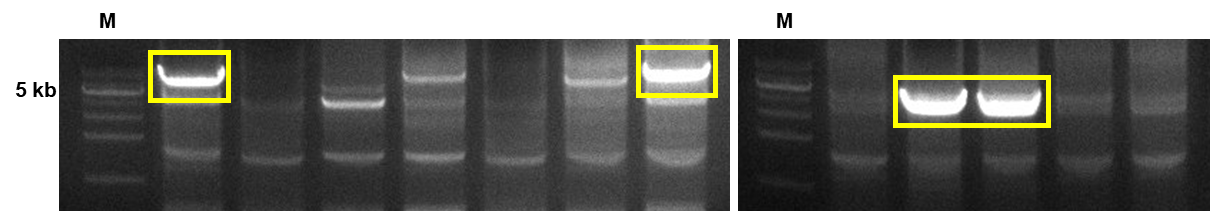


**Supplementary Figure 6**. Target-specific DNA insertion (6.4 kb) conjugated with CRISPR-Cas9. PCR of DNA extracted from pale green colonies of *CrFTSY* knock-out *C*. *reinhardtii*. 4 positive colonies of 12 candidates were clearly confirmed. M, 1 kb DNA ladder.
