# Supplementary material for: Site-Specific Gene Knock-Out and On-Site Heterologous Gene Overexpression in Chlamydomonas reinhardtii via a CRISPR-Cas9-Mediated Knock-in Method
Source: Front Plant Sci. 2020 Mar 20;11:306. doi: 10.3389/fpls.2020.00306 (PMC7099044; doi:10.3389/fpls.2020.00306)

Supplementary Material 2

**Raw data of sequencing**

**CreFTSY_KI_gLuciferase gene + Hygromycin resistance gene**


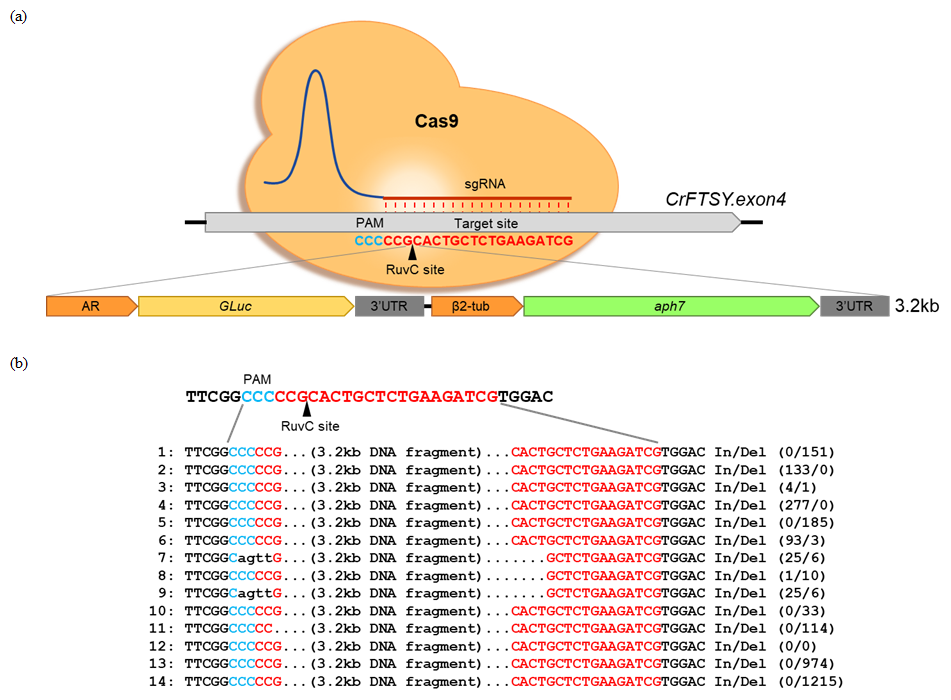

CrFTSY-Ga mutant 1

Primer_F


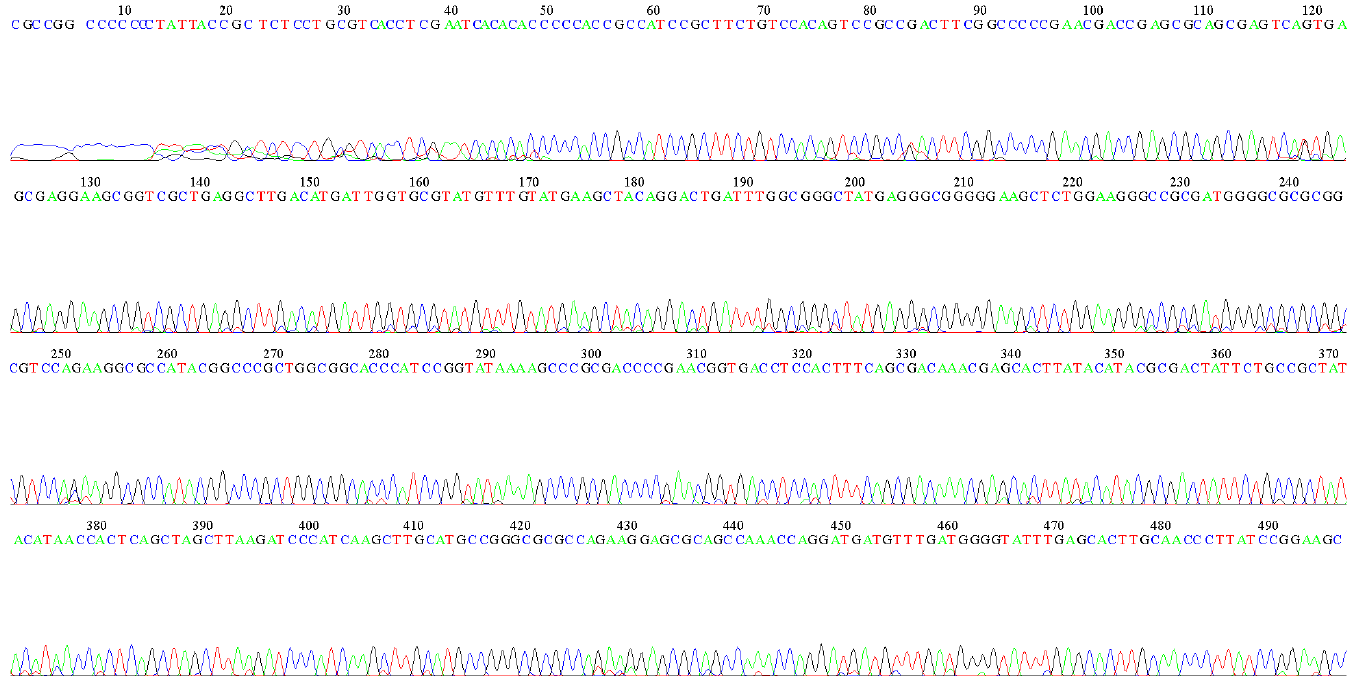


Primer_R


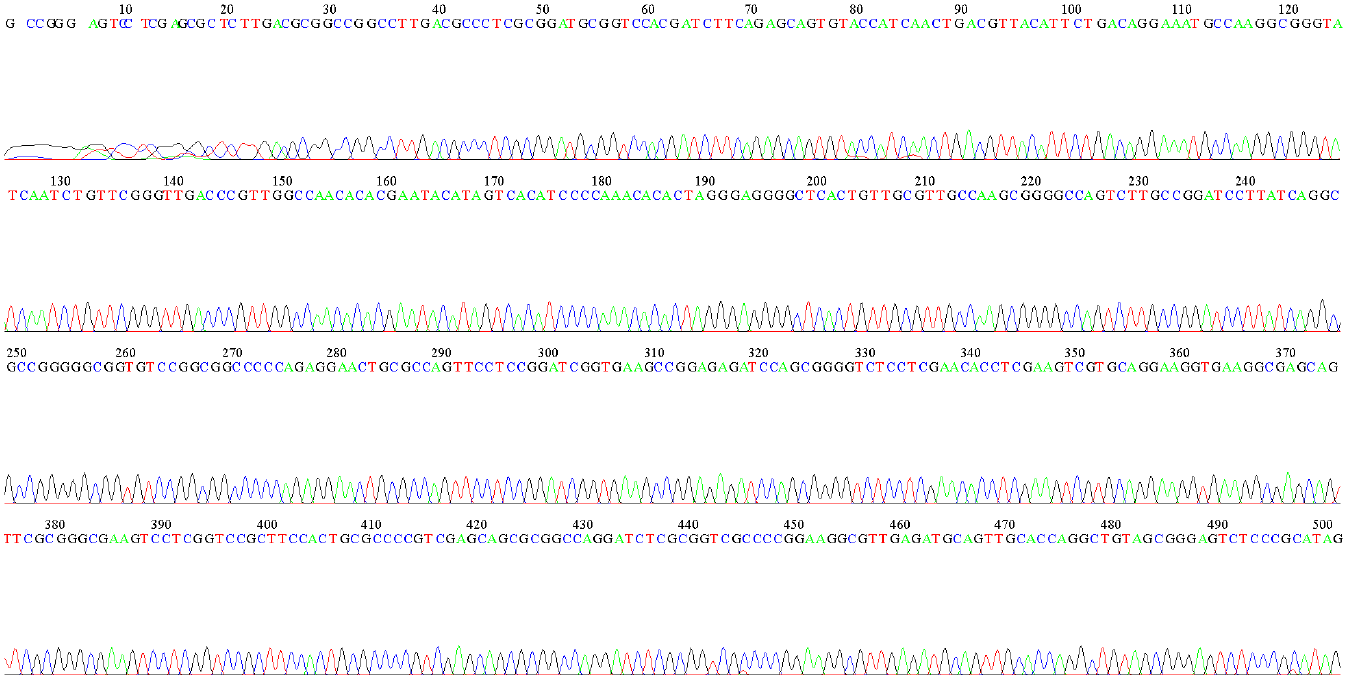


CrFTSY-Ga mutant 2

Primer_F


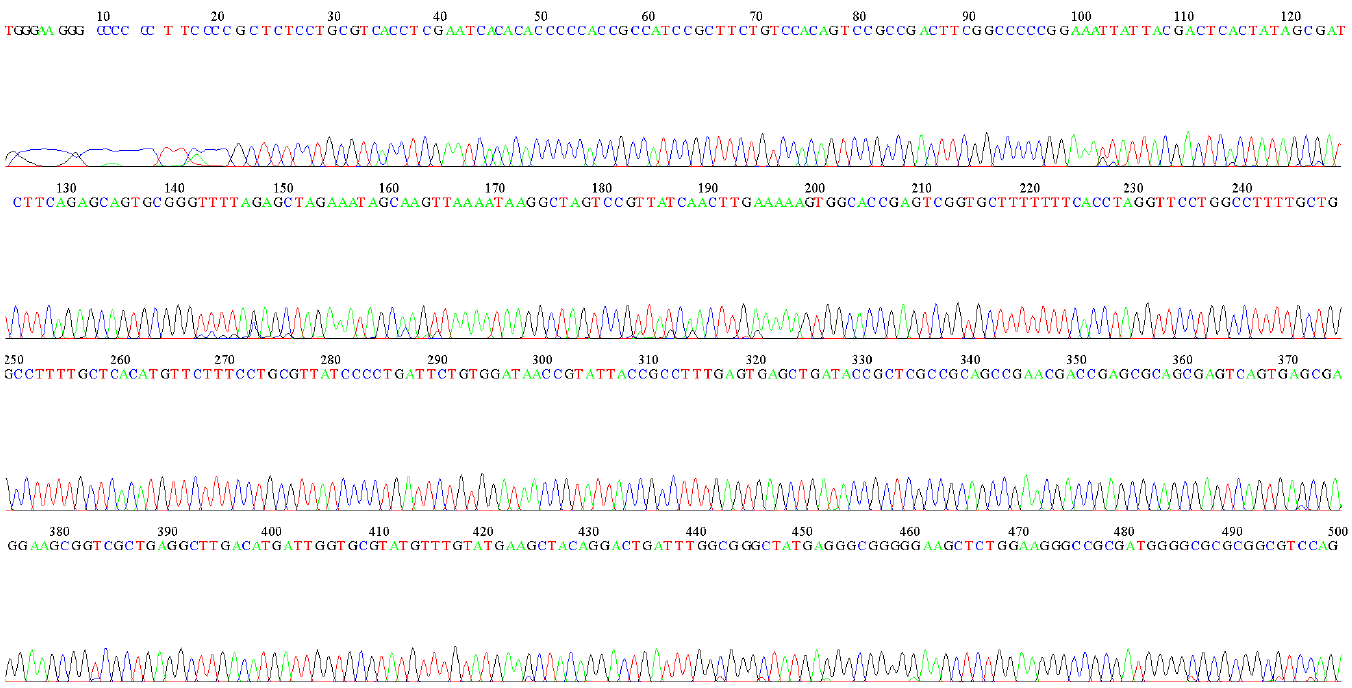


Primer_R


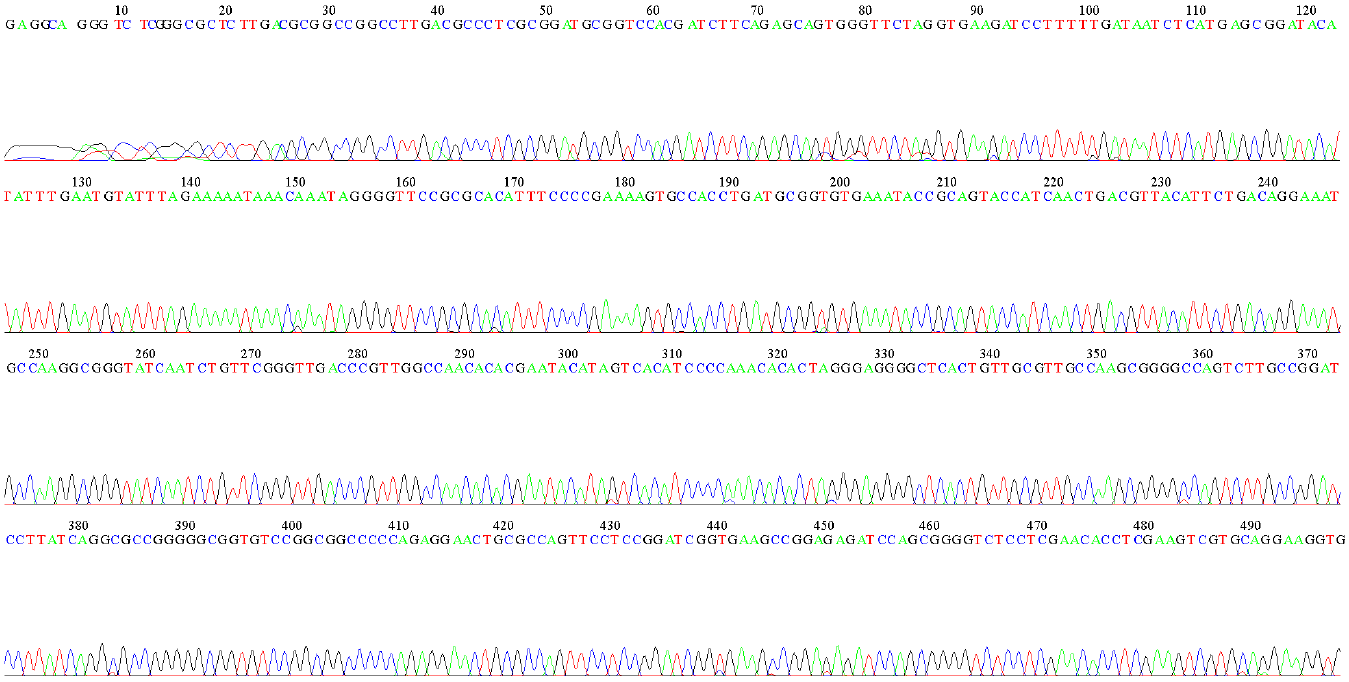


CrFTSY-Ga mutant 3

Primer_F


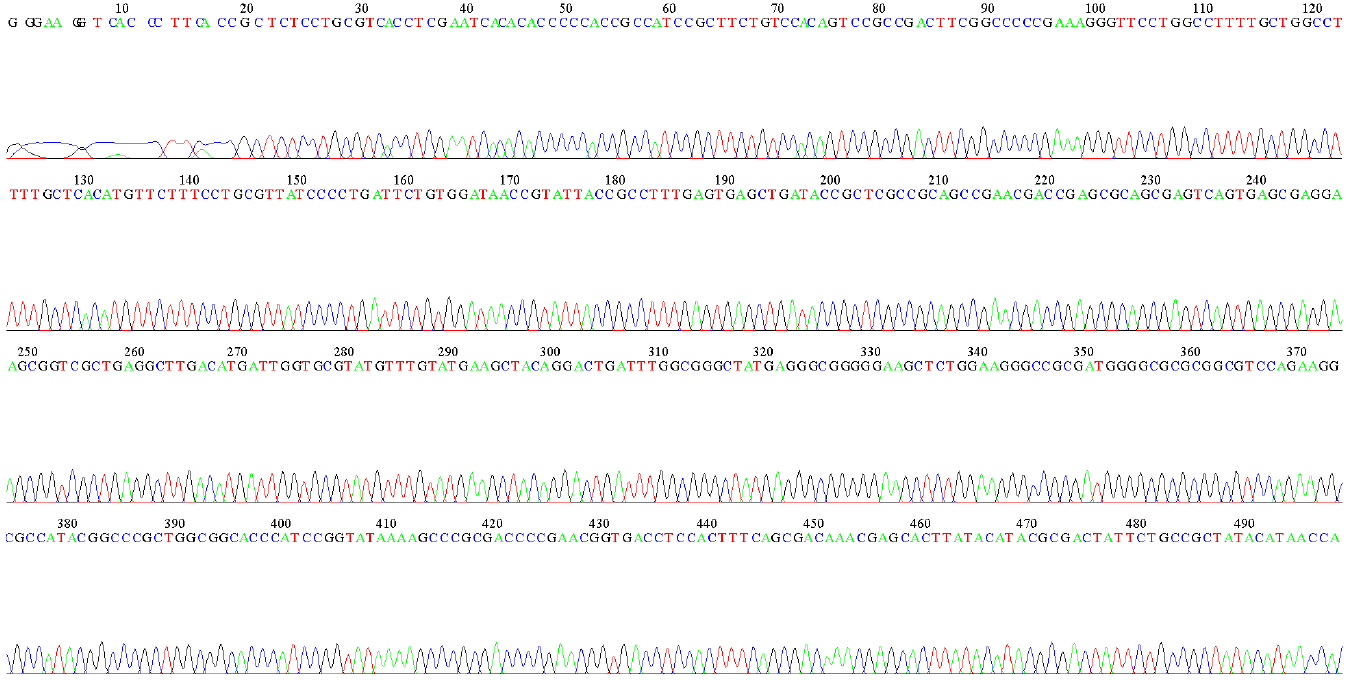


Primer_R


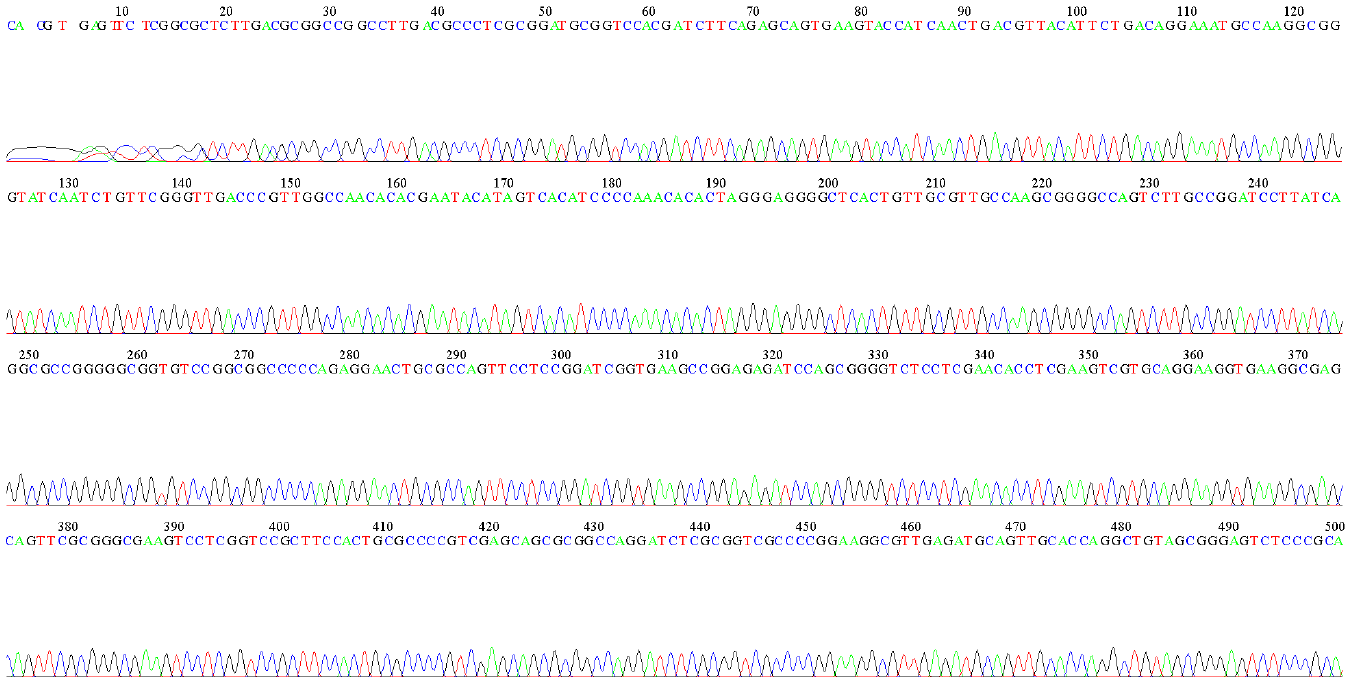


CrFTSY-Ga mutant 4

Primer_F


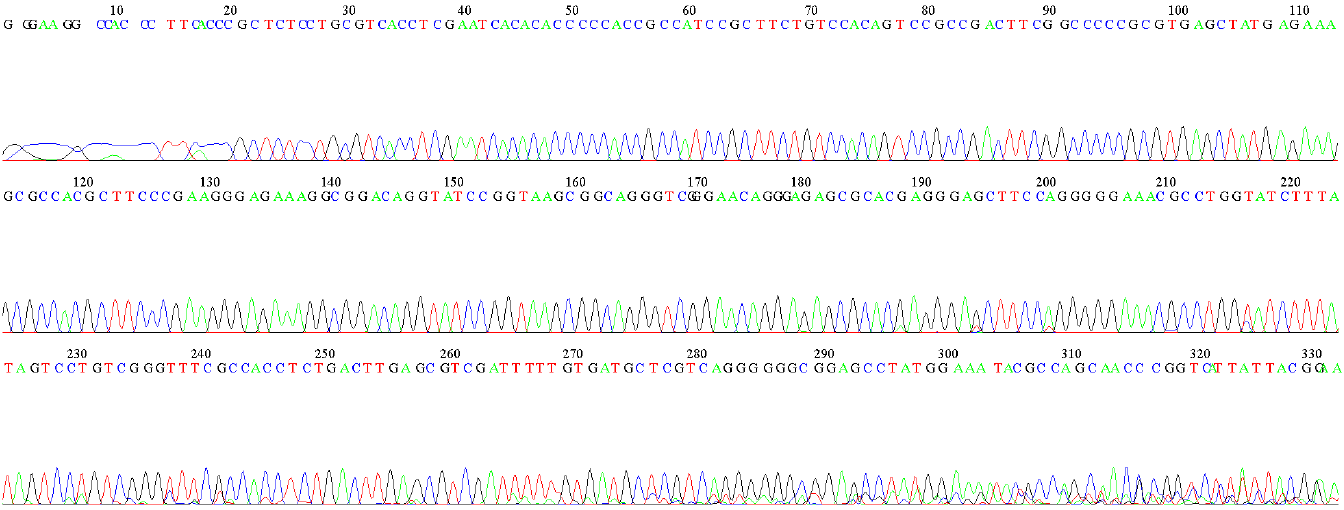


Primer_R


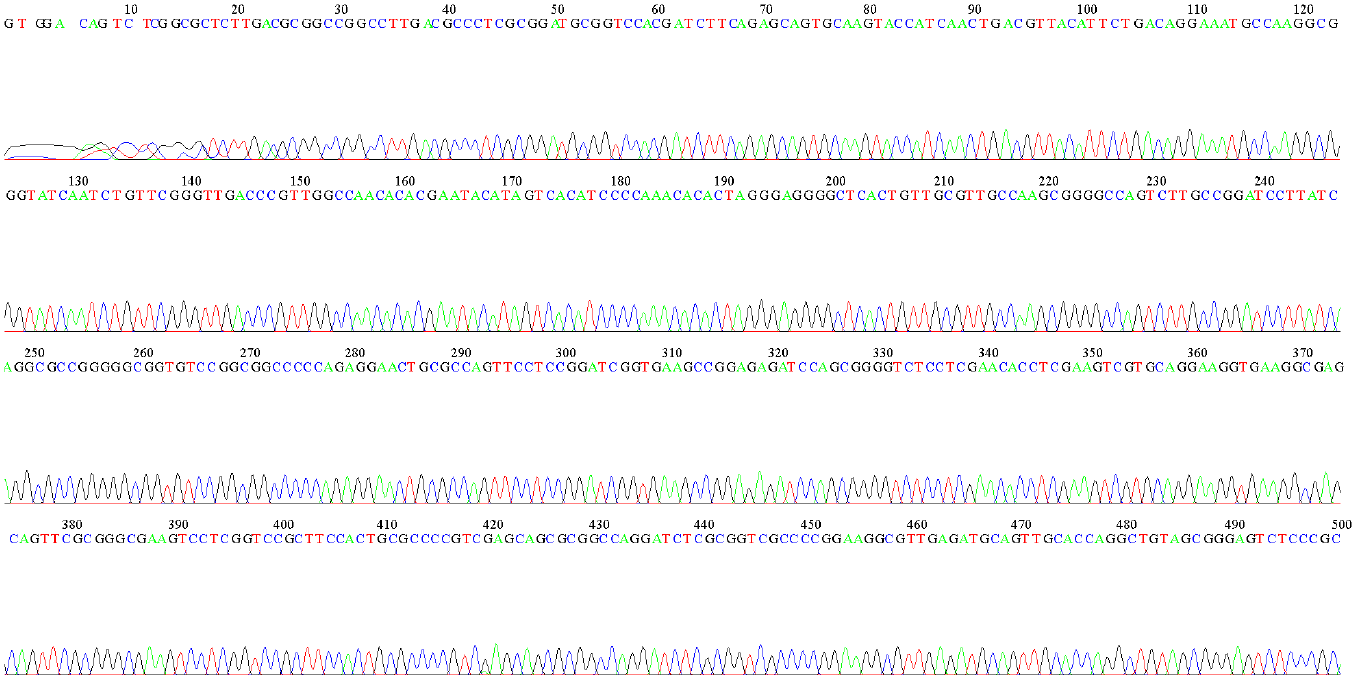


CrFTSY-Ga mutant 5

Primer_F


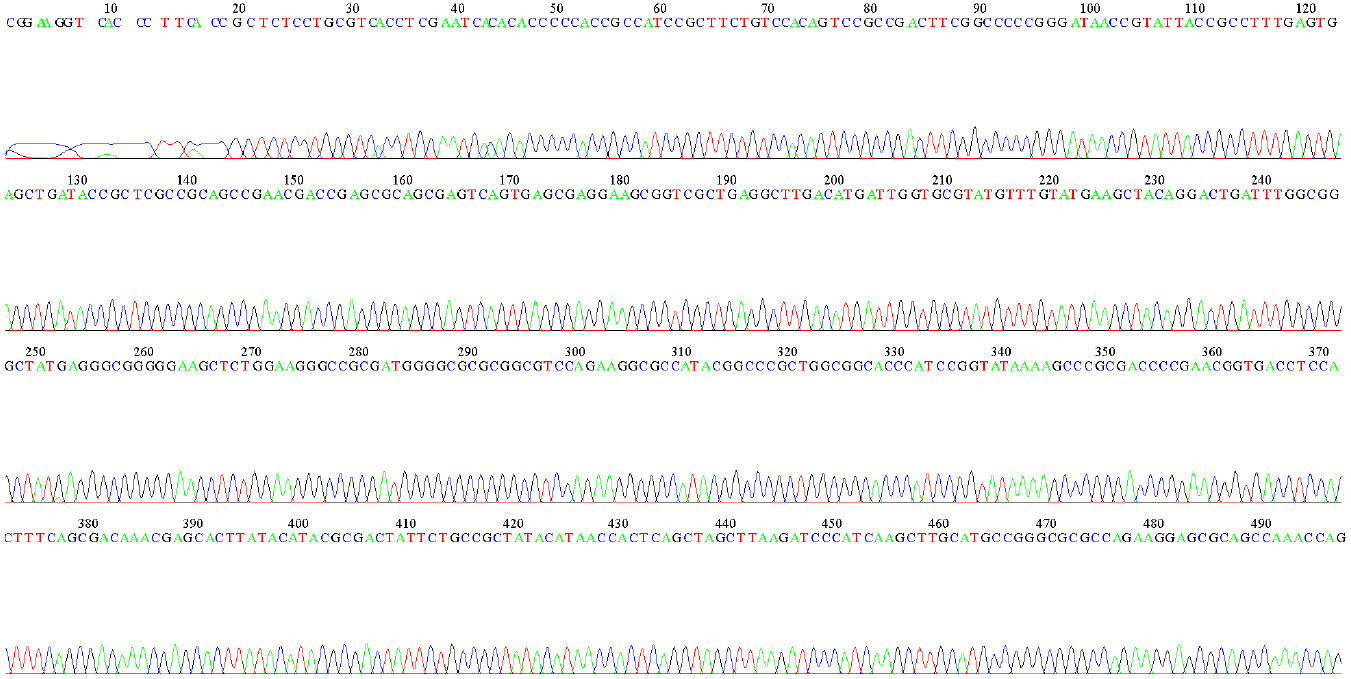


Primer_R


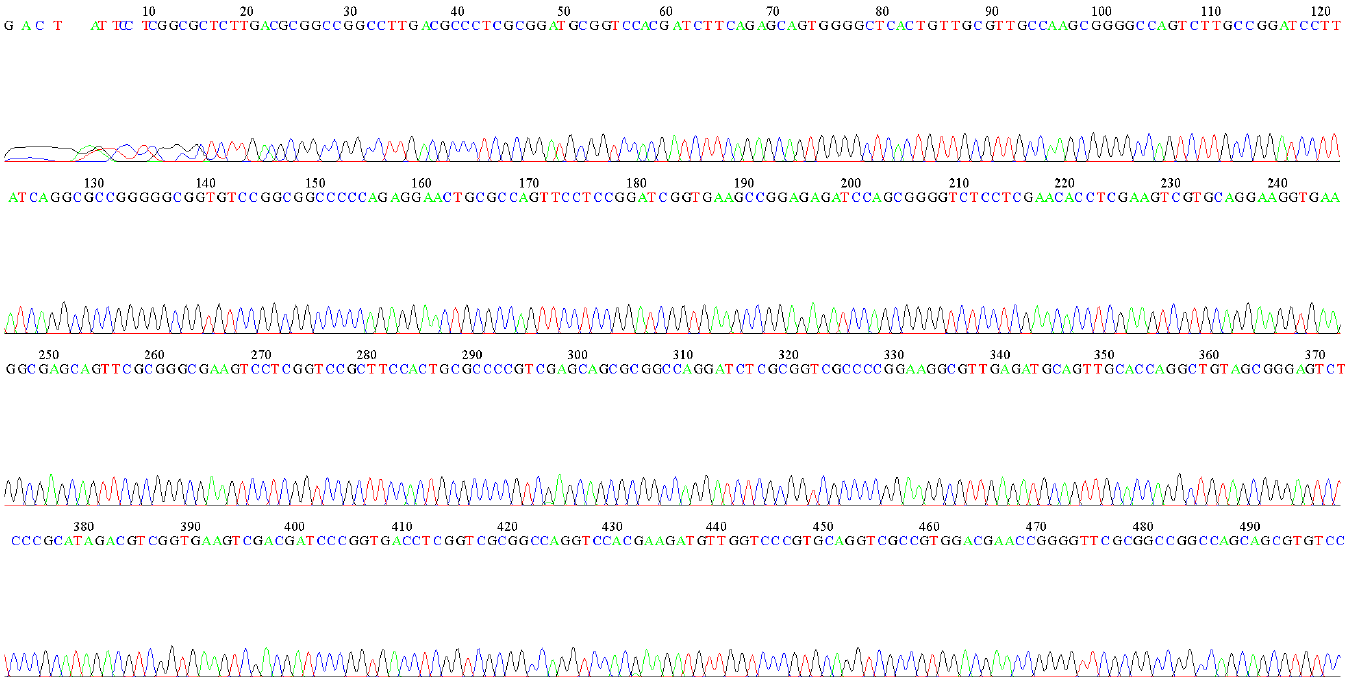


CrFTSY-Ga mutant 6

Primer_F


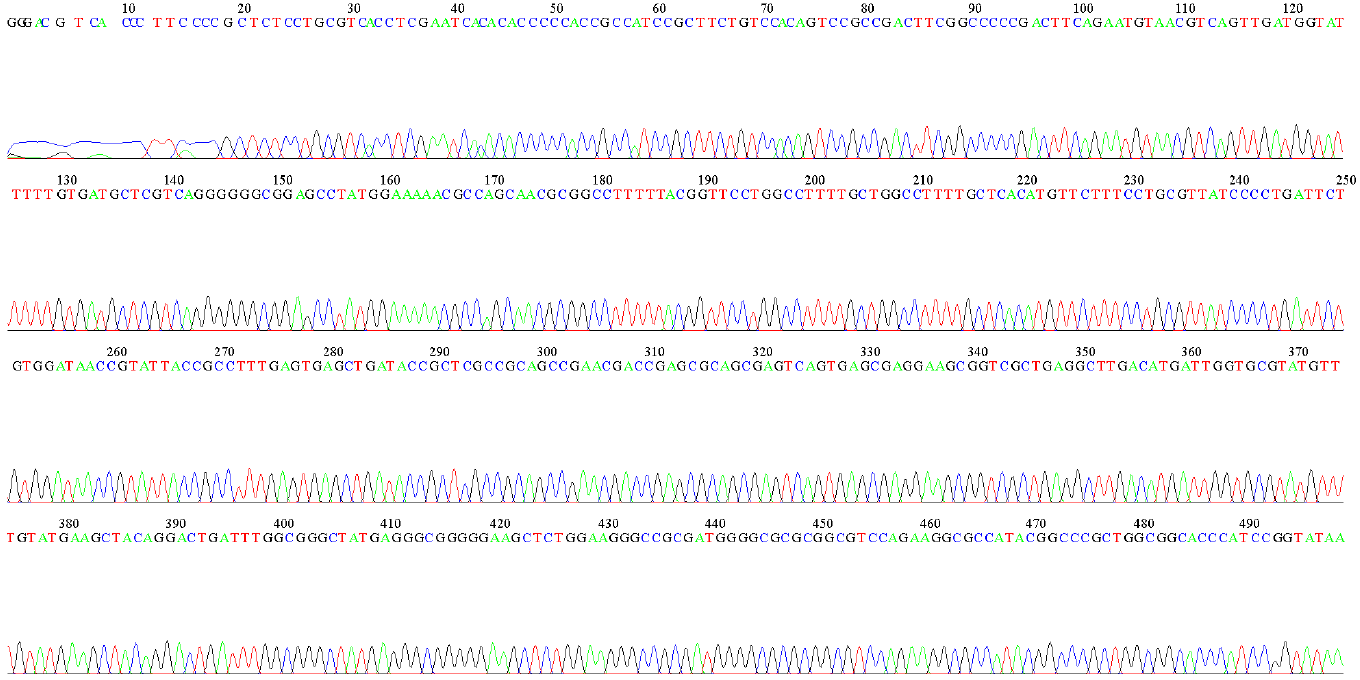


Primer_R


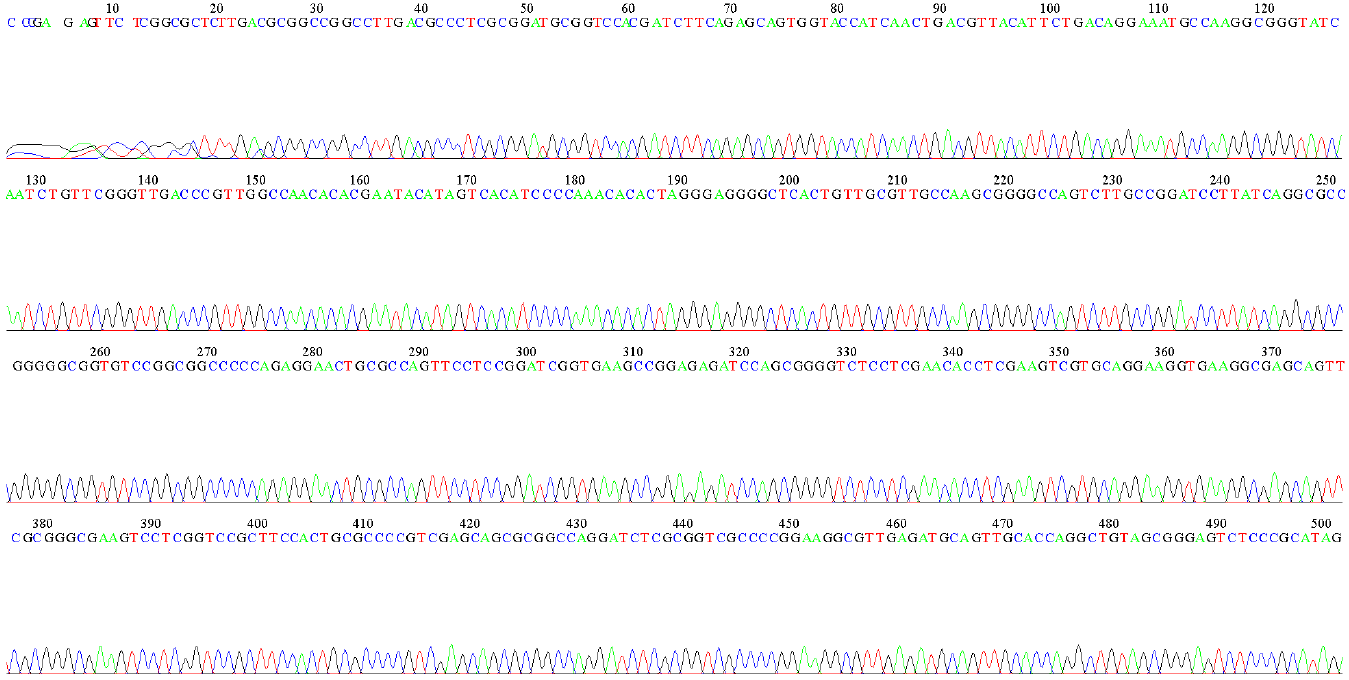


CrFTSY-Ga mutant 7

Primer_F


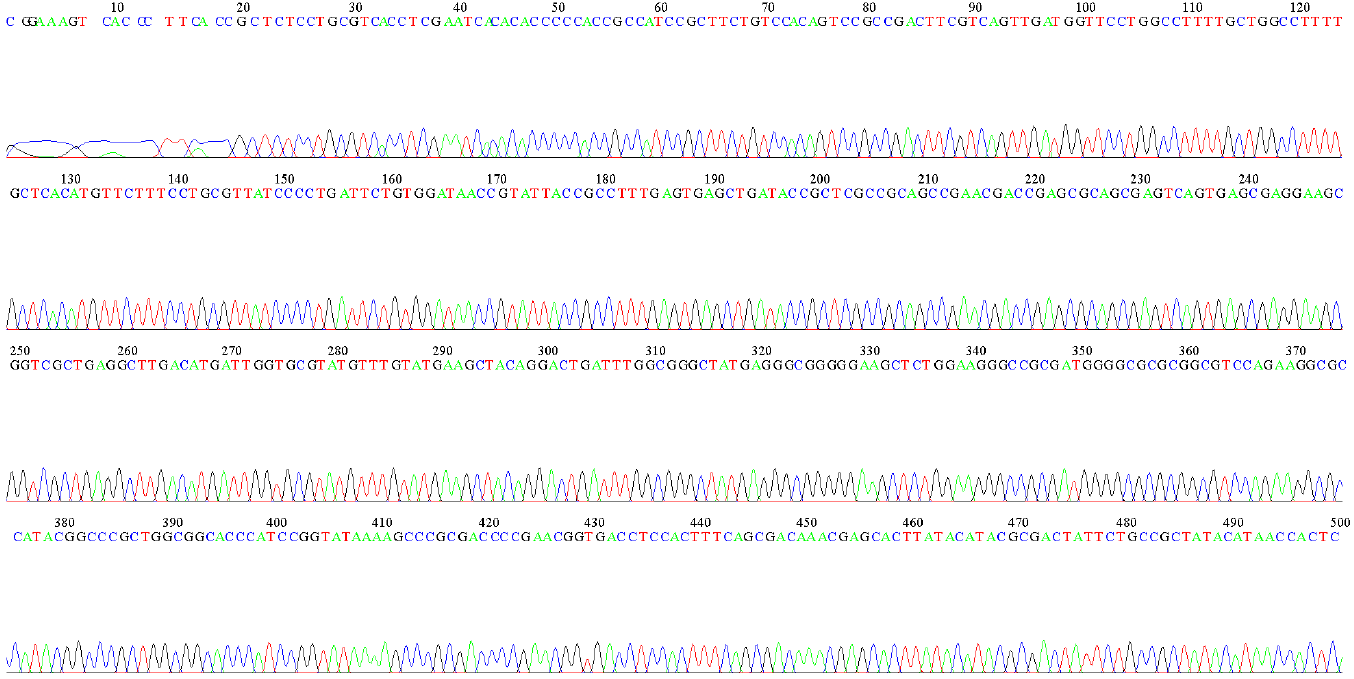


Primer_R


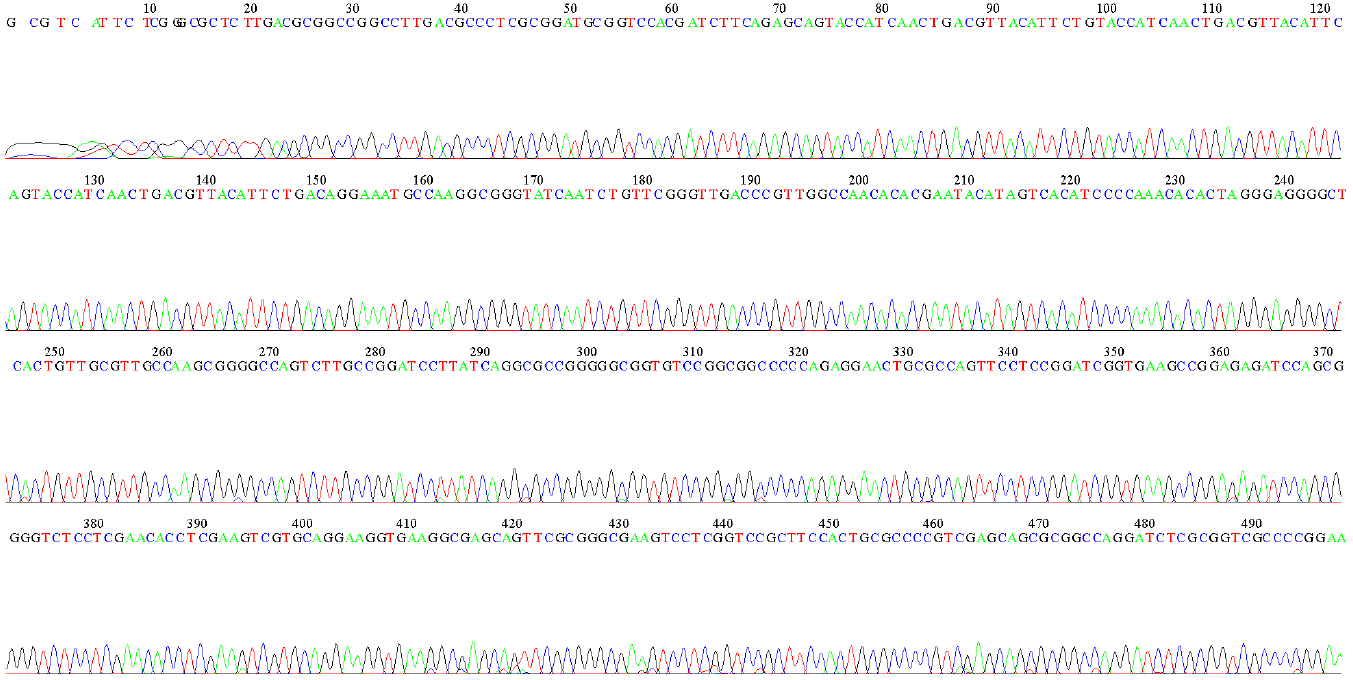


CrFTSY-Ga mutant 8

Primer_F


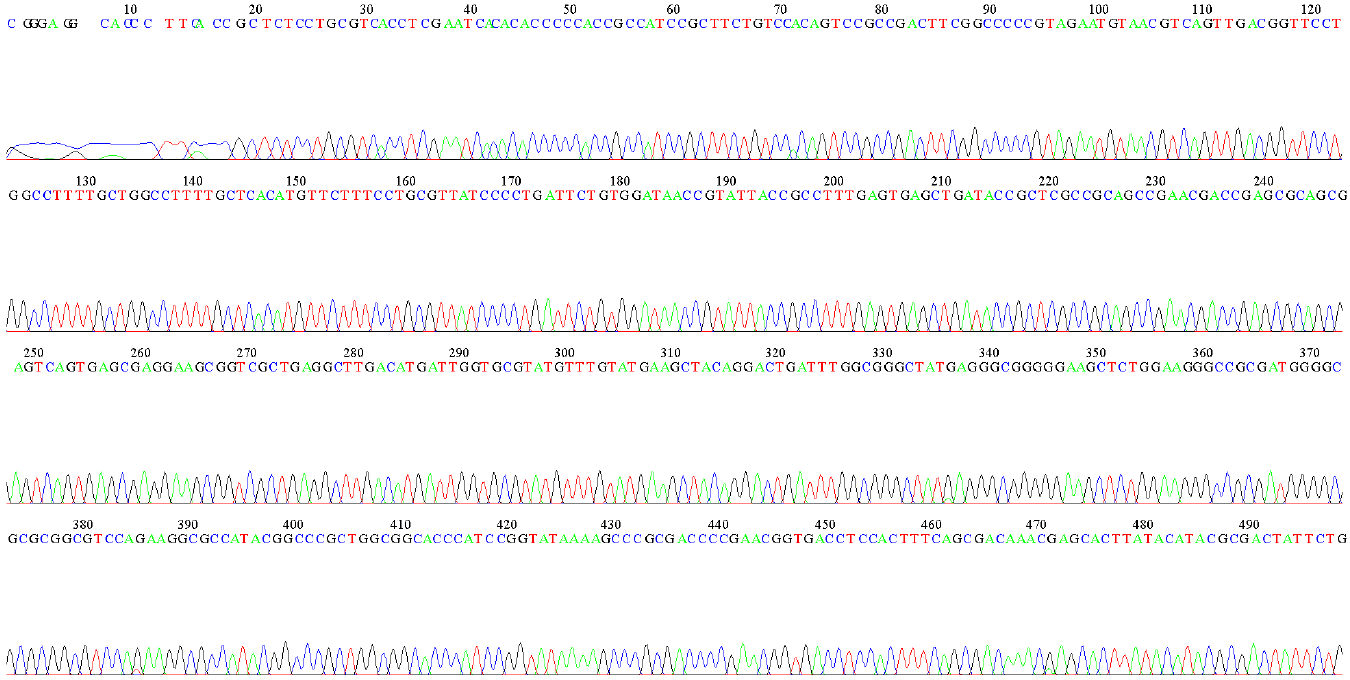


Primer_R


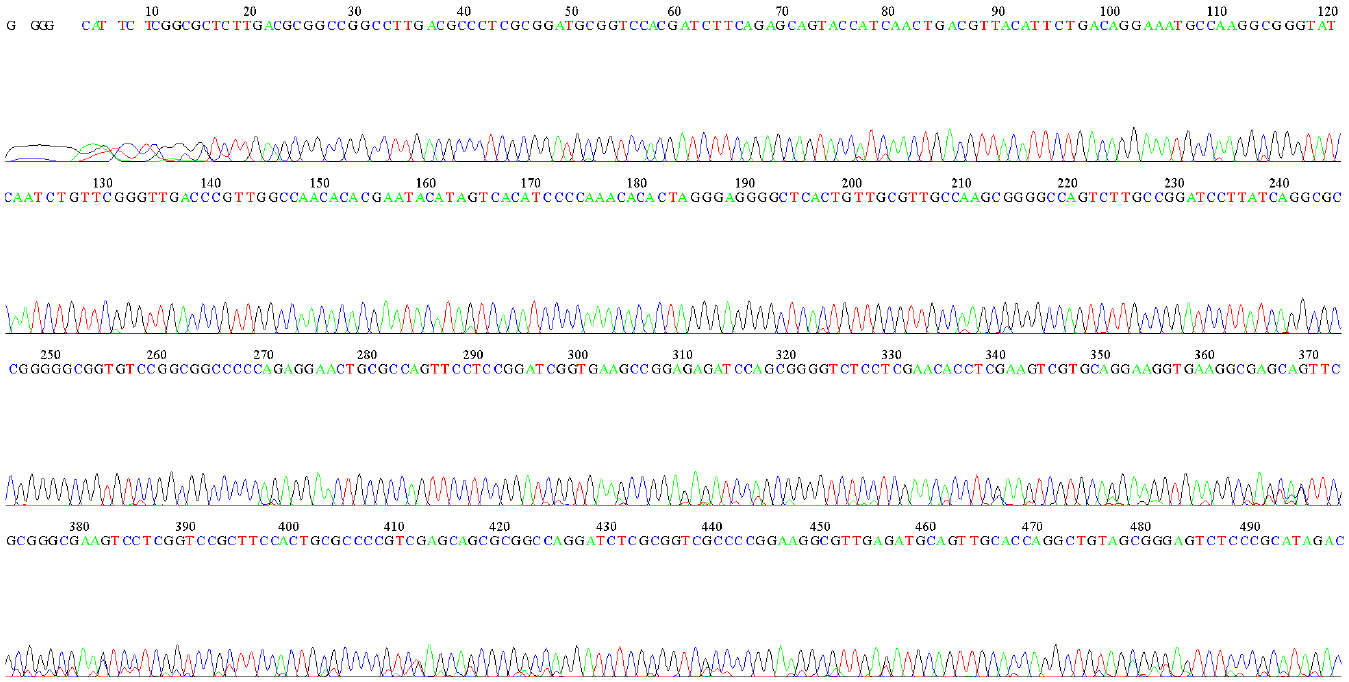


CrFTSY-Ga mutant 9

Primer_F


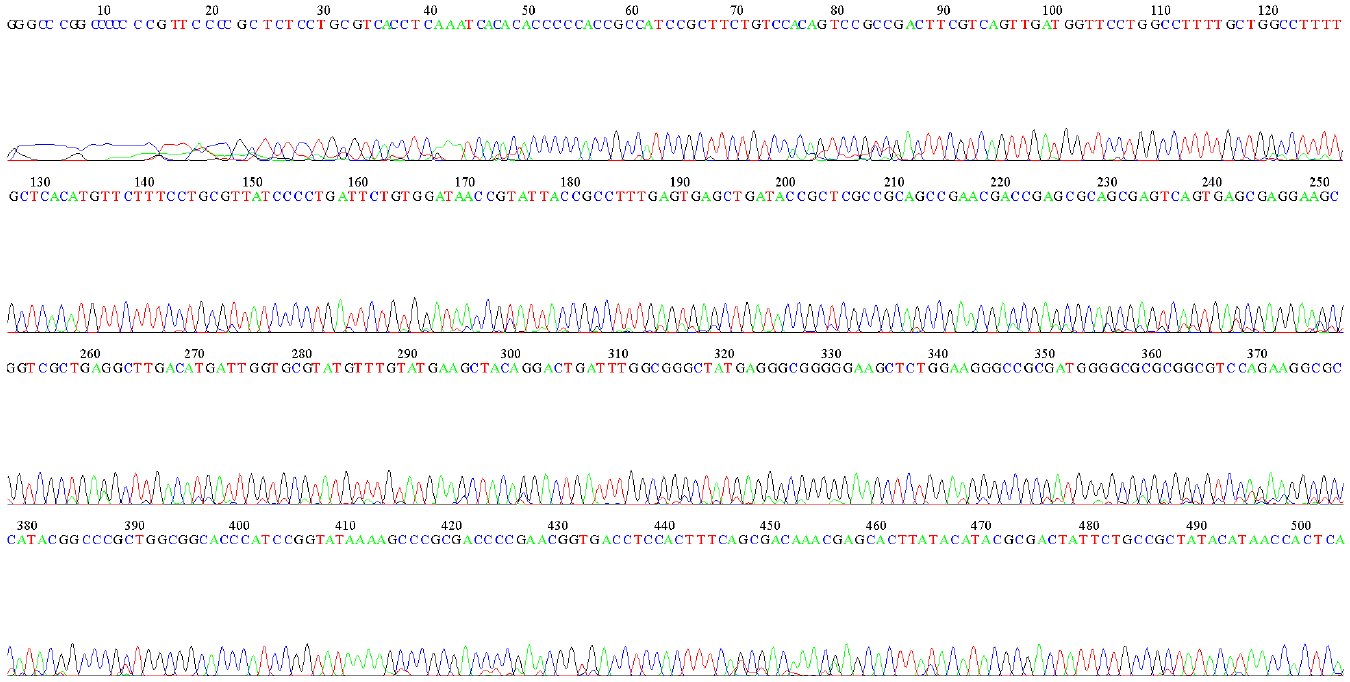


Primer_R


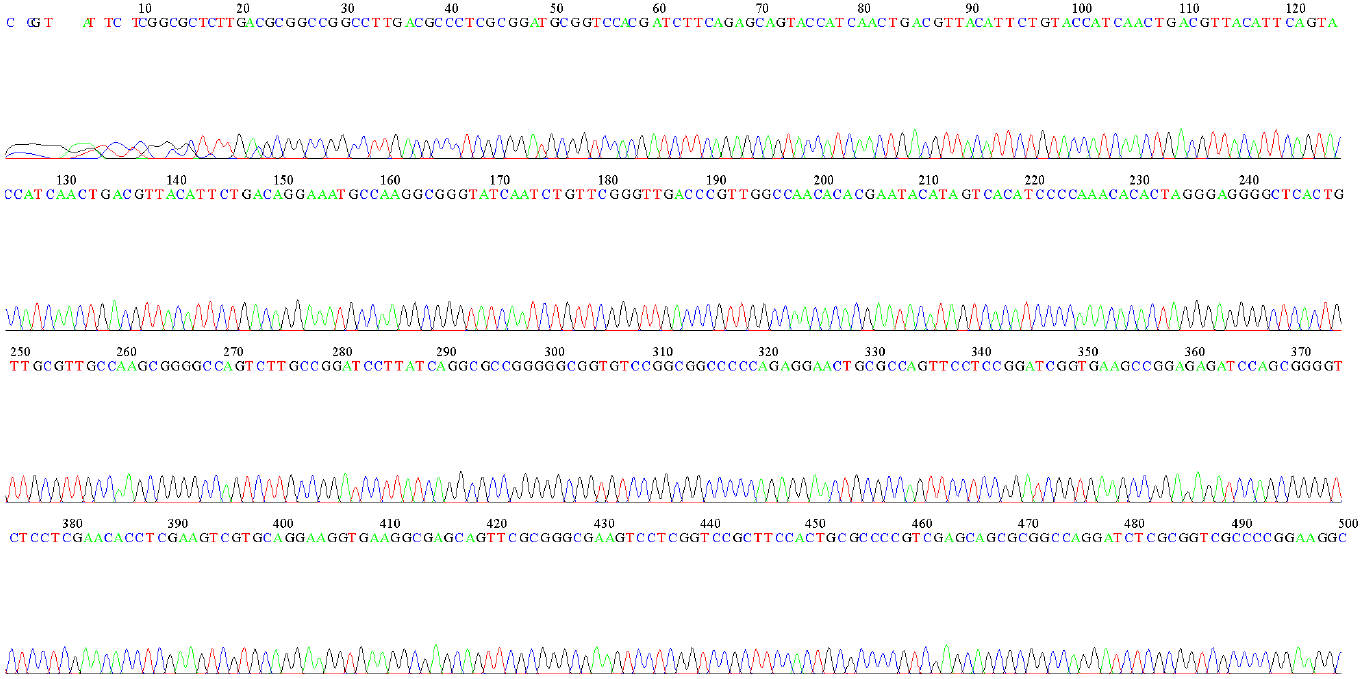


CrFTSY-Ga mutant 10

Primer_F


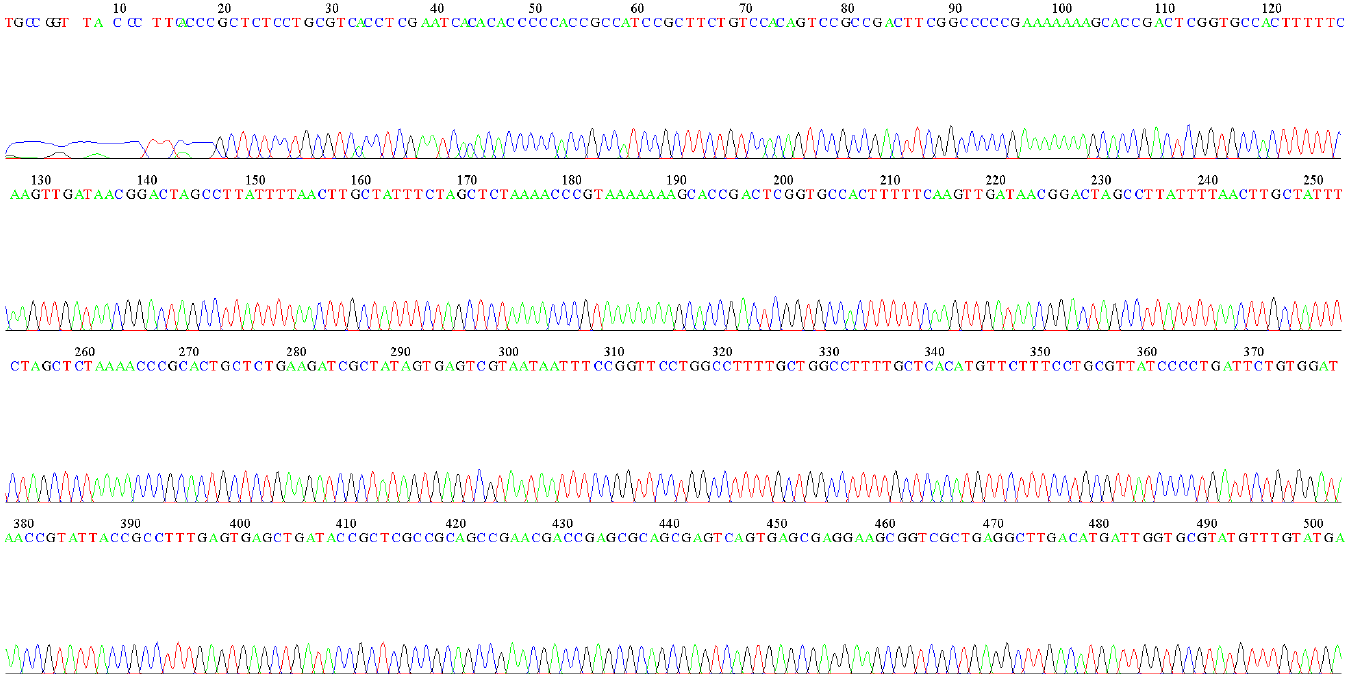


Primer_R


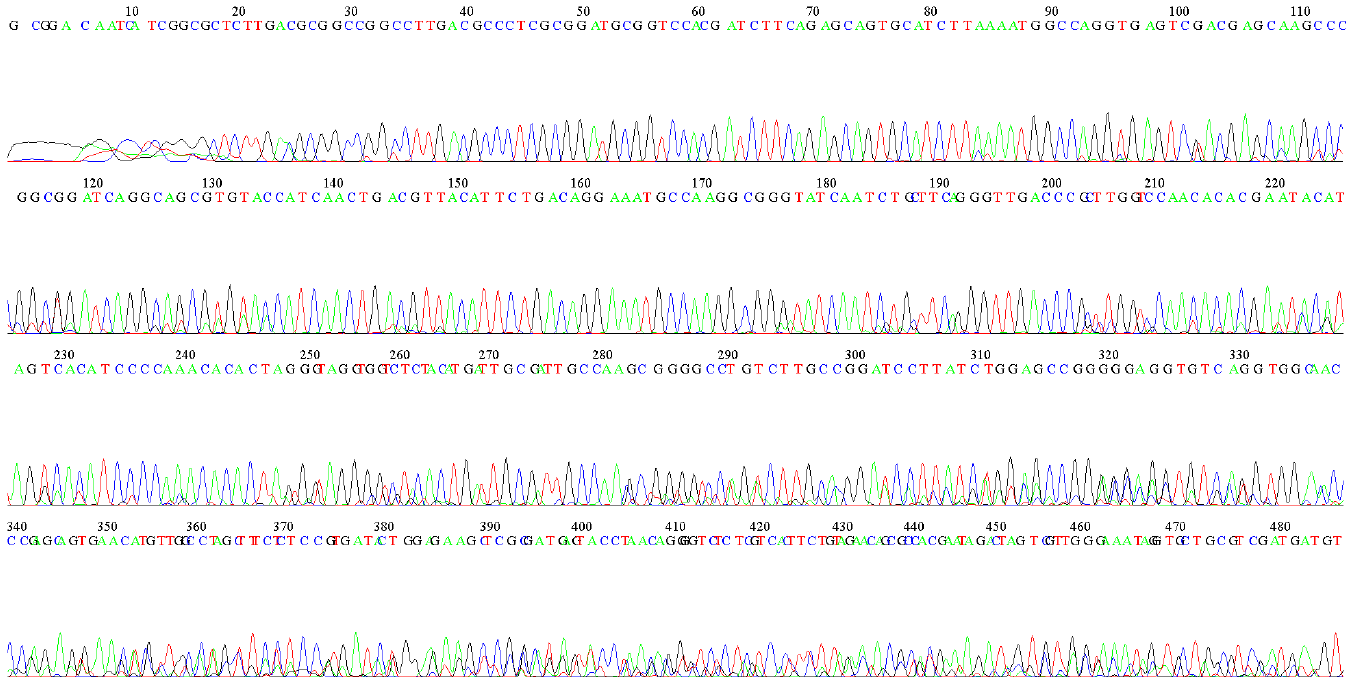


CrFTSY-Ga mutant 11

Primer_F


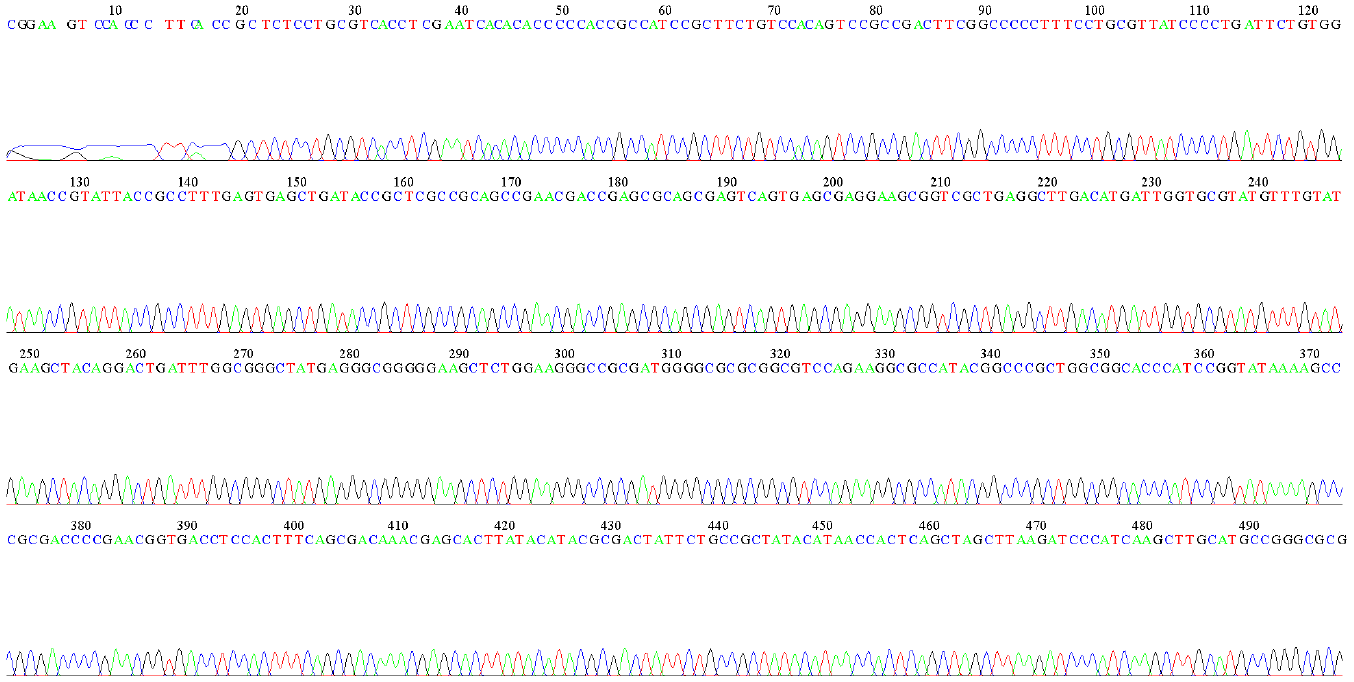


Primer_R


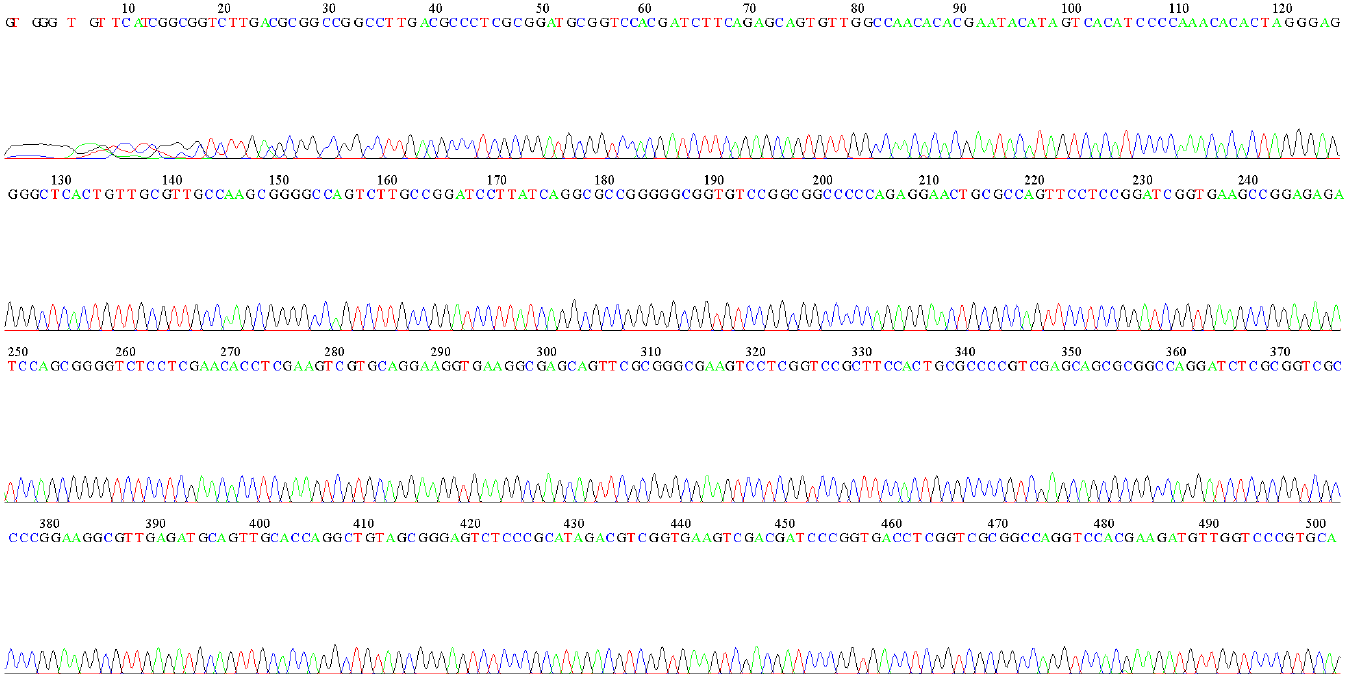


CrFTSY-Ga mutant 12

Primer_F


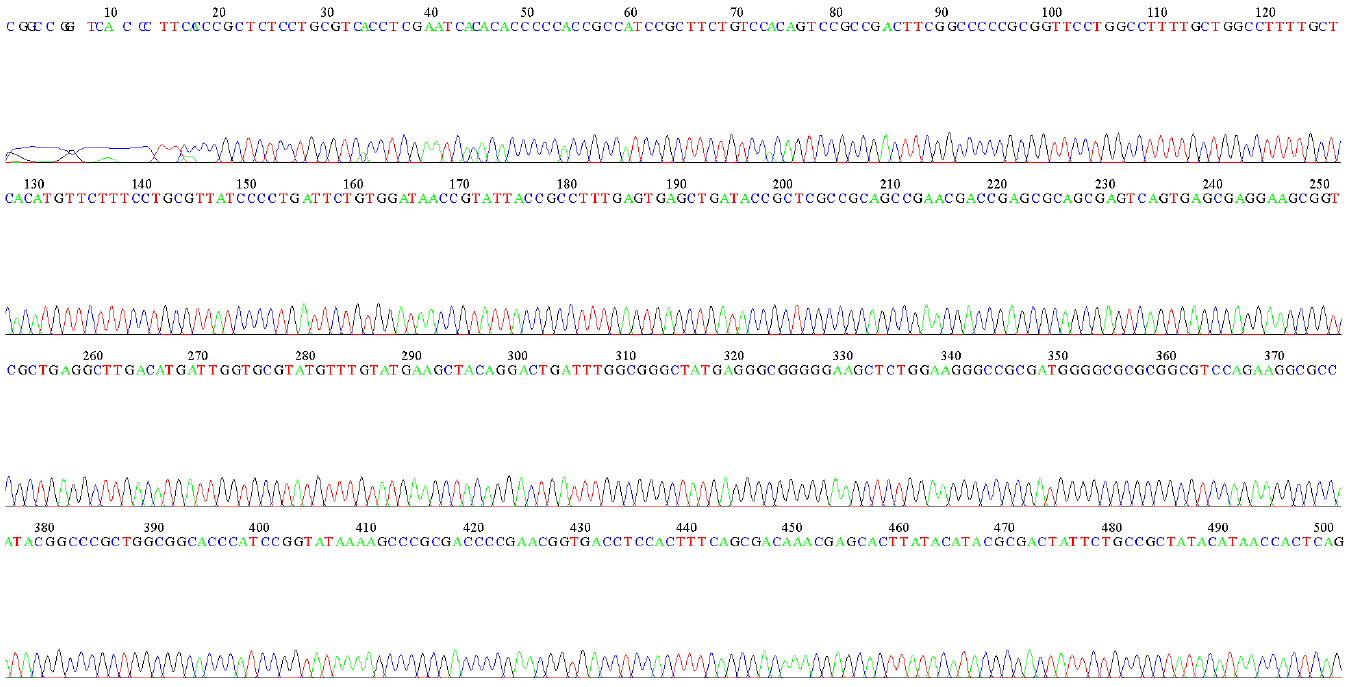


Primer_R


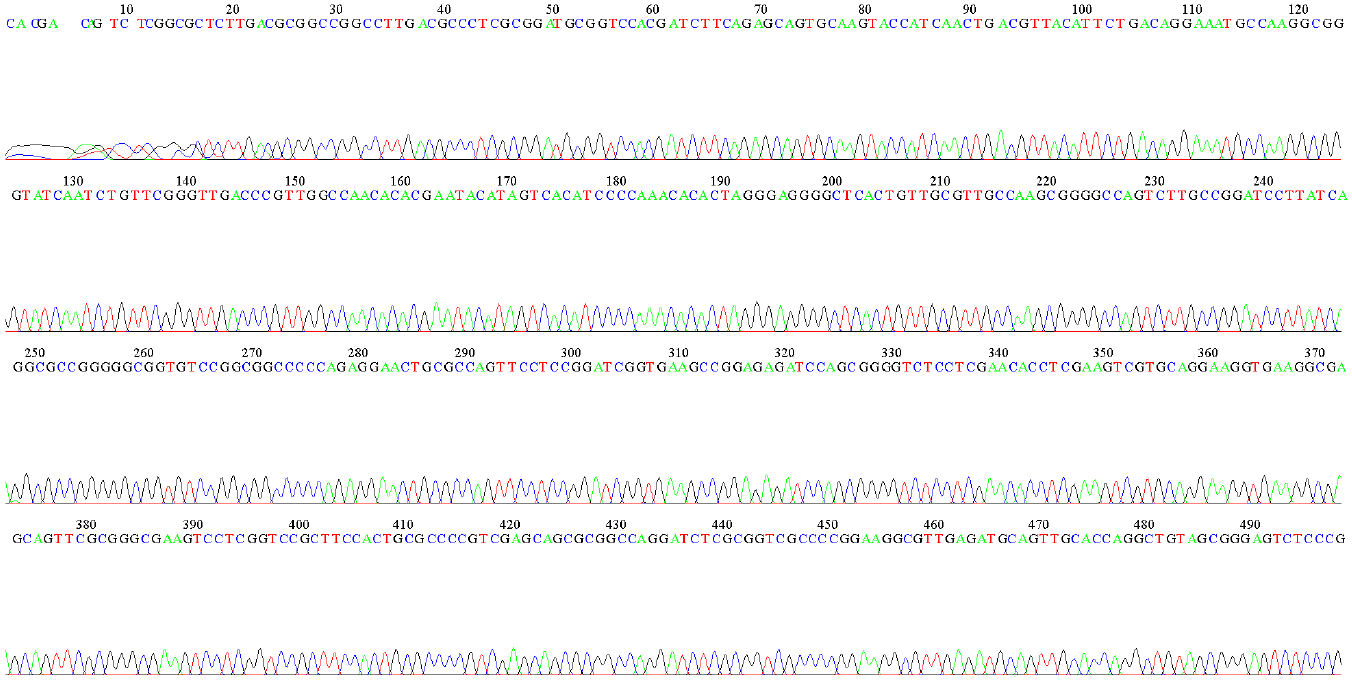


CrFTSY-Ga mutant 13

Primer_F


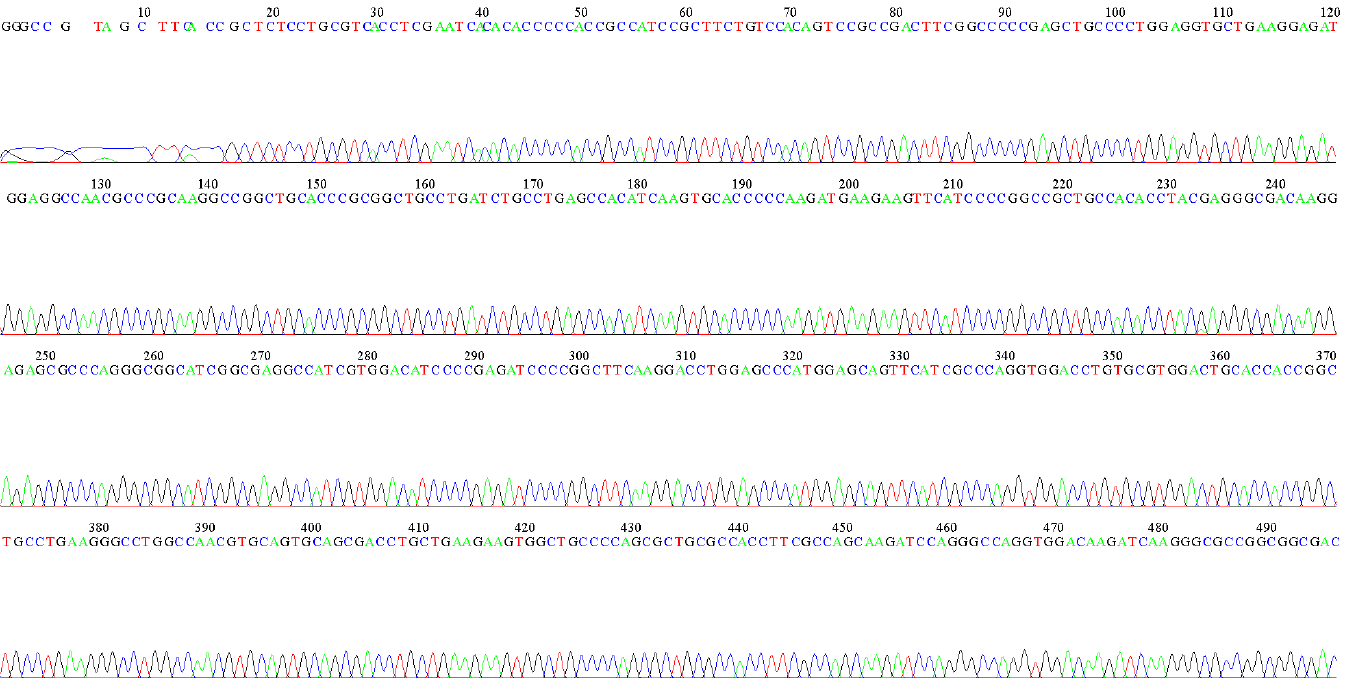


Primer_R


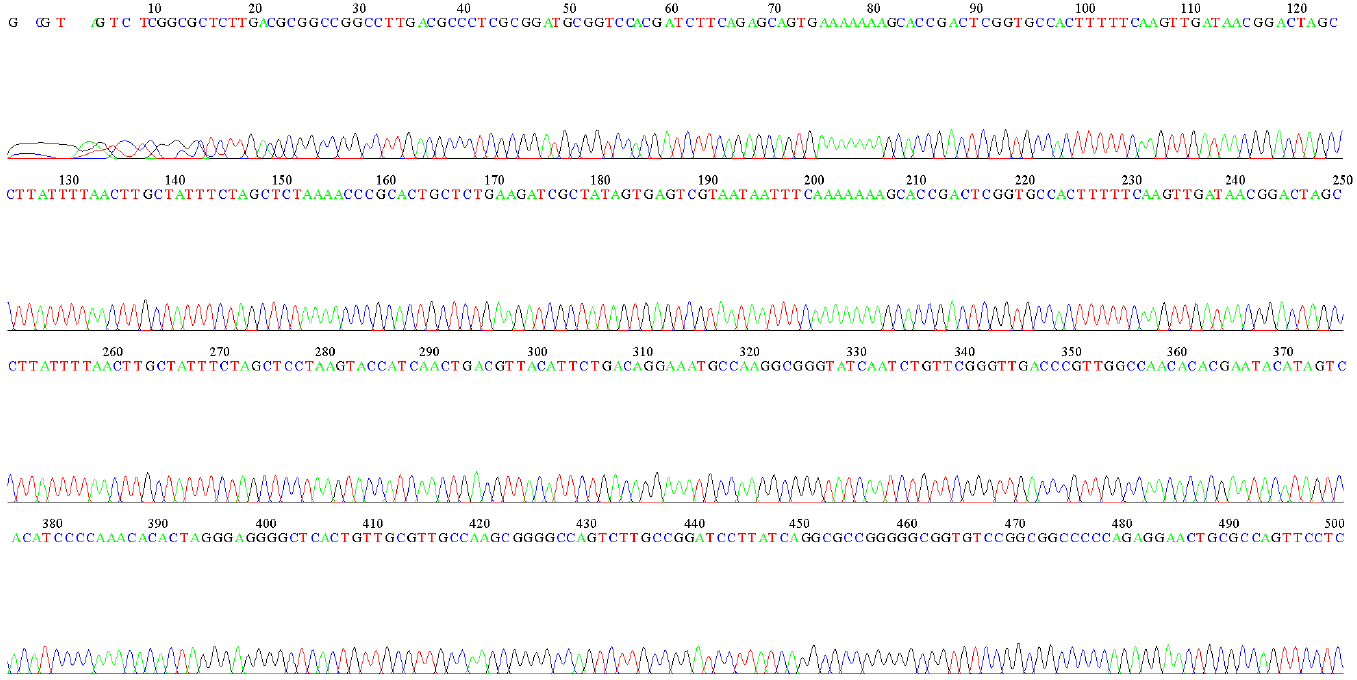


CrFTSY-Ga mutant 14

Primer_F


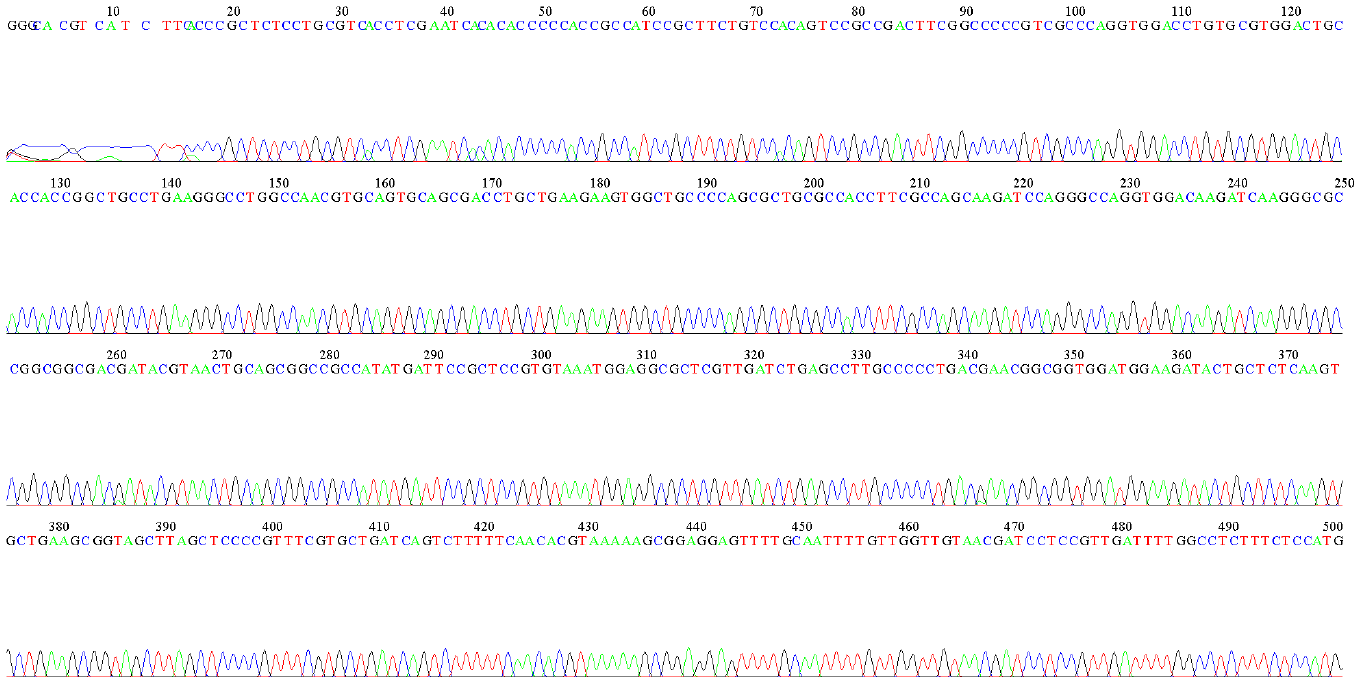


Primer_R


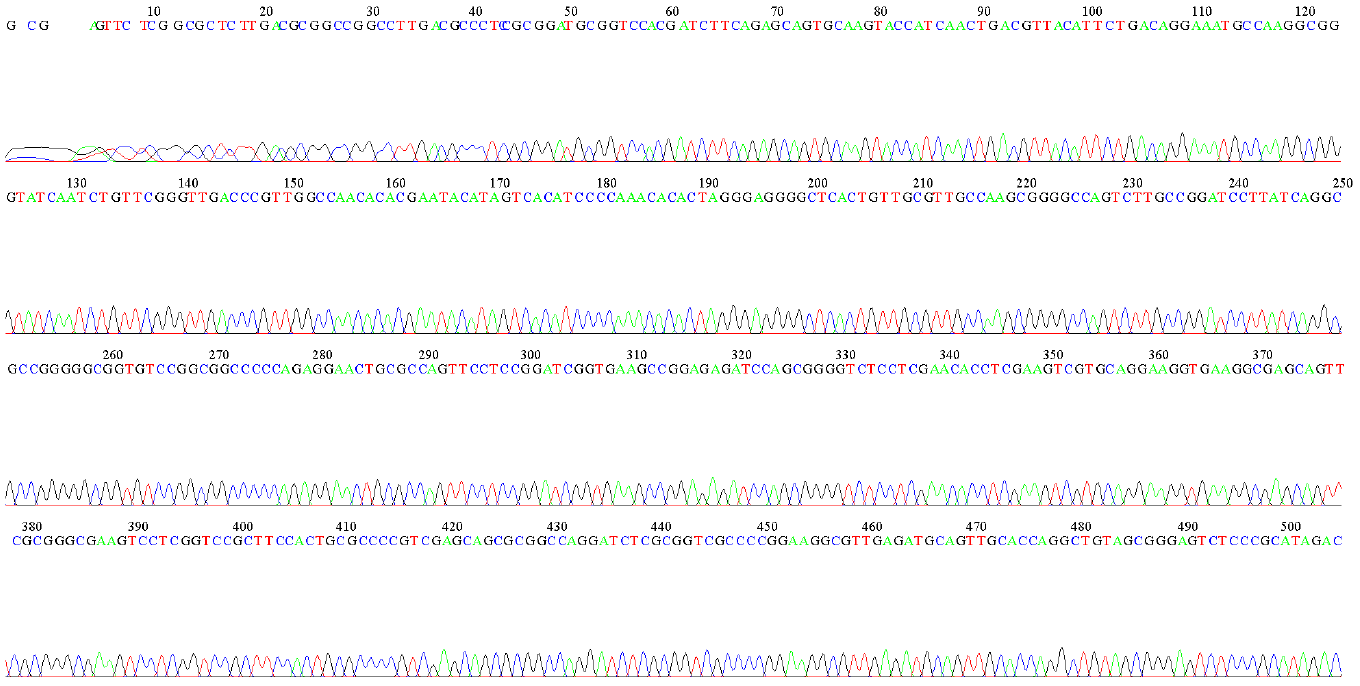


**CreFTSY_KI_Paromomycin resistance gene + mVenus gene + Hygromycin resistance gene**


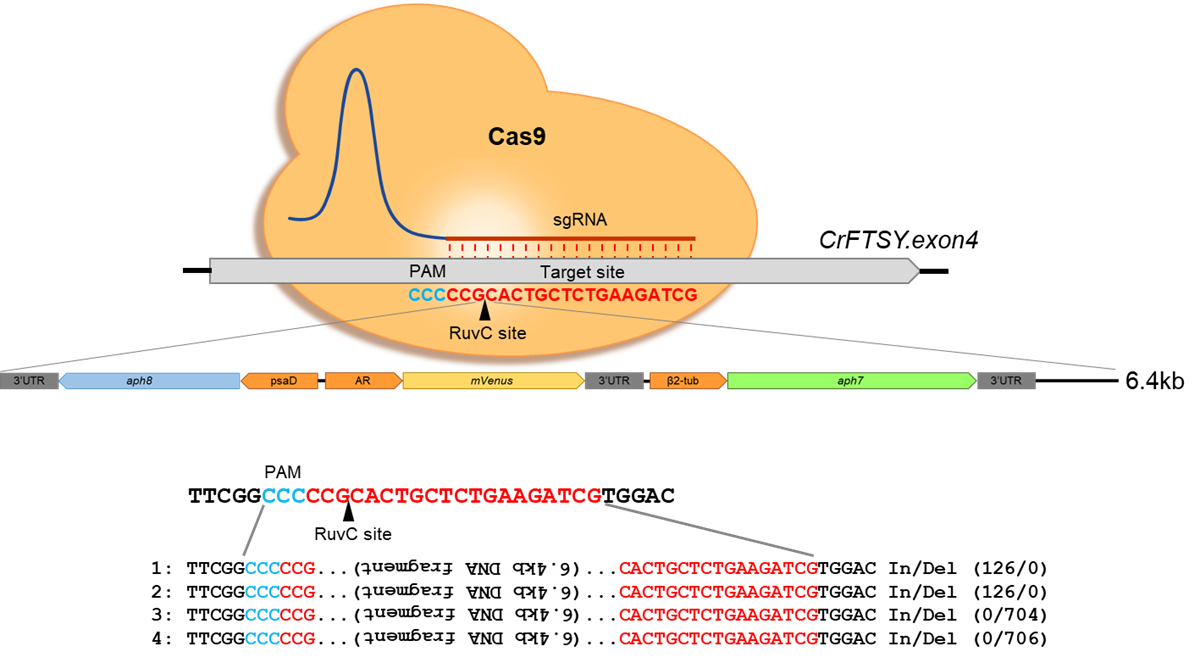


**
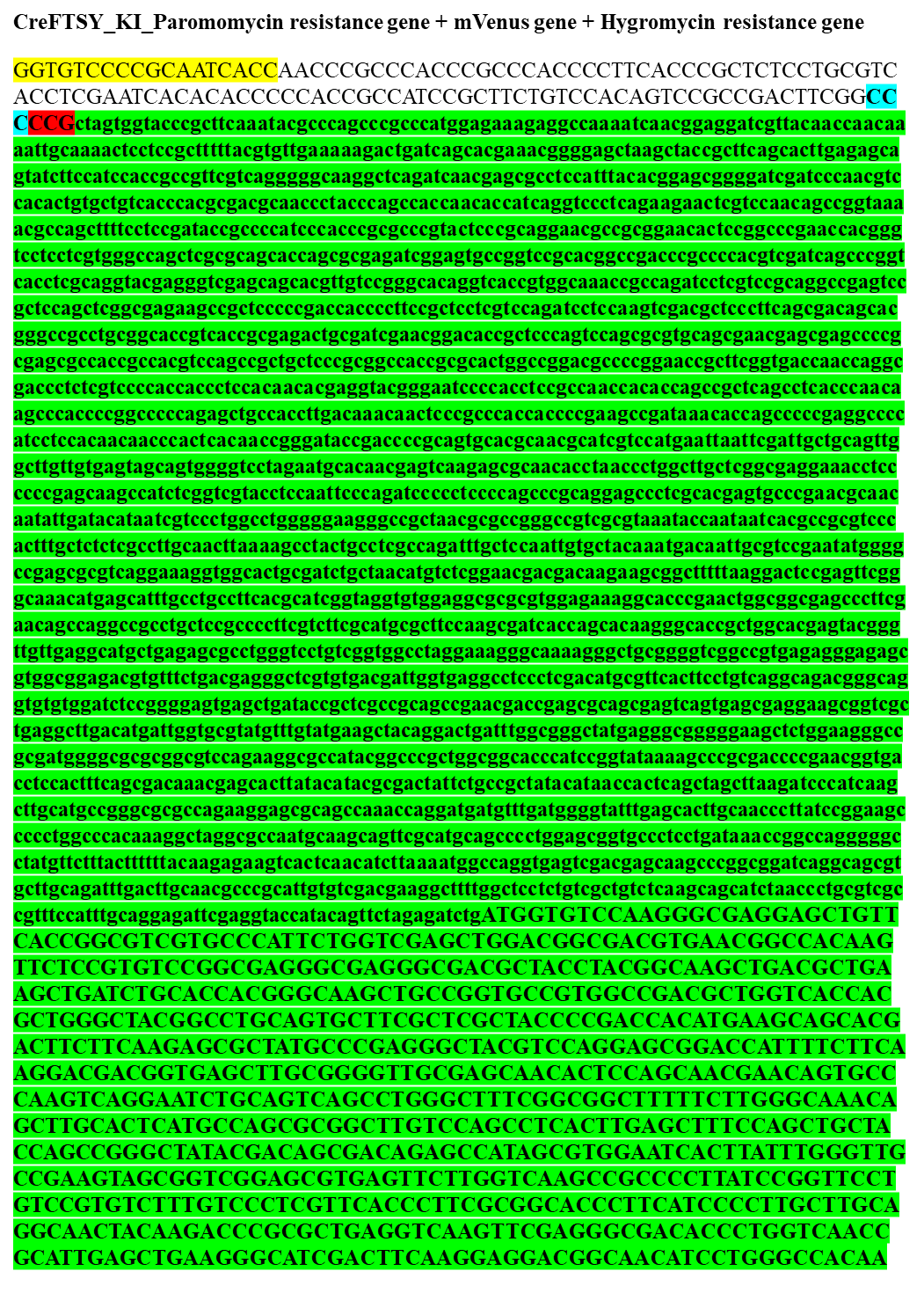
**


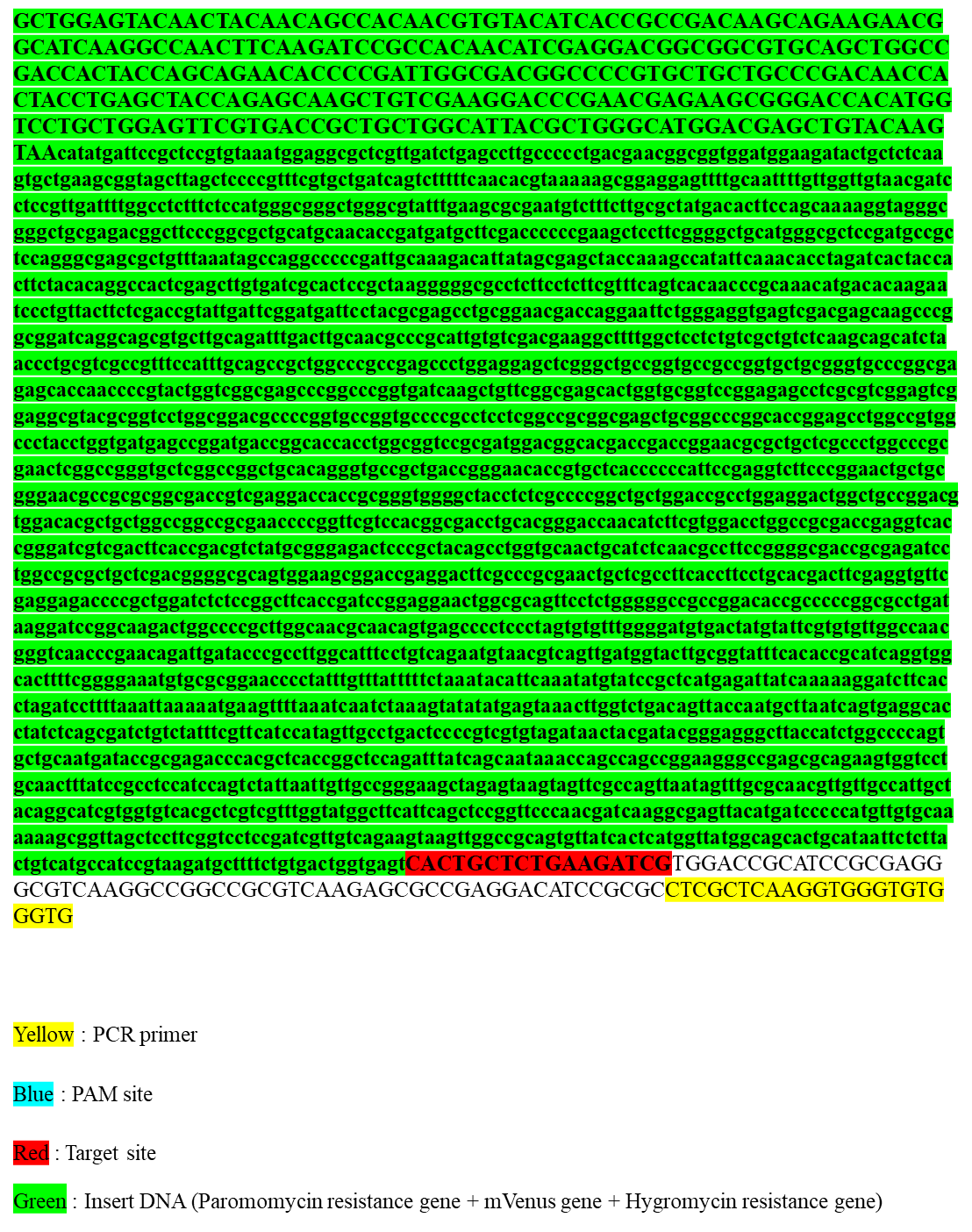


ΔCrFTSY_mV mutant 1

Primer_F


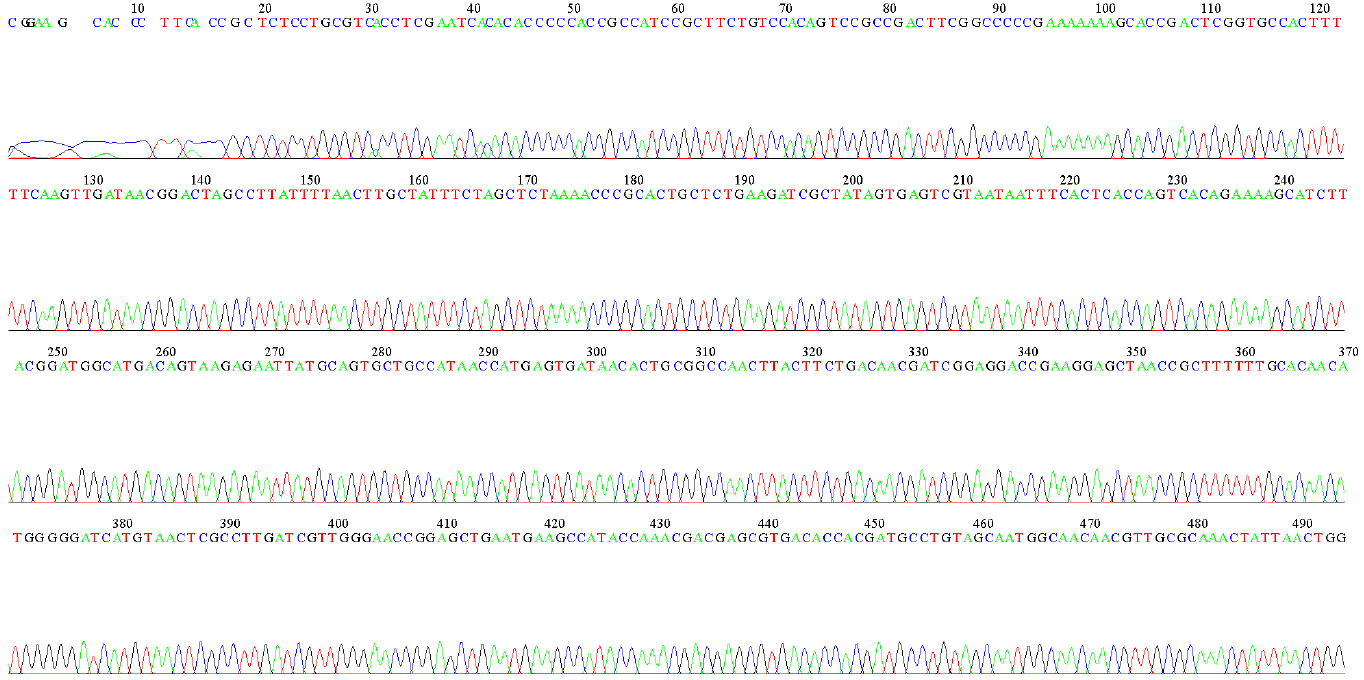


Primer_R


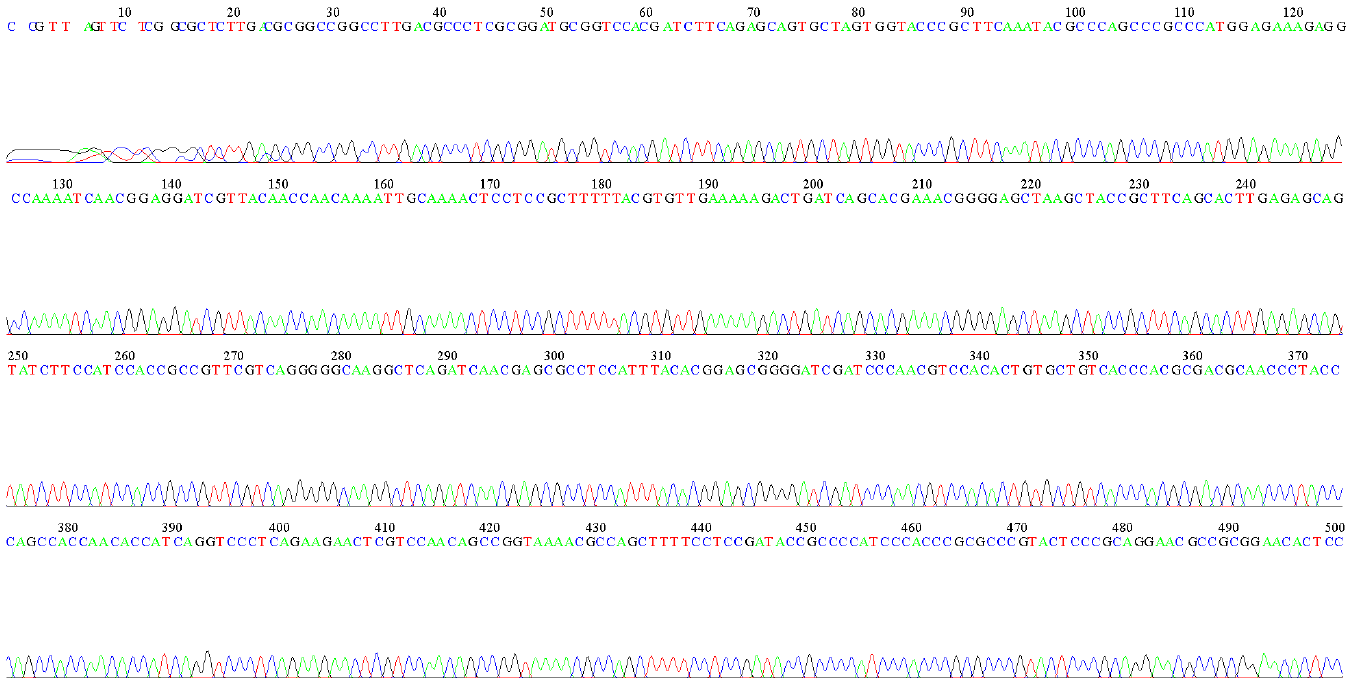


ΔCrFTSY_mV mutant 2

Primer_F


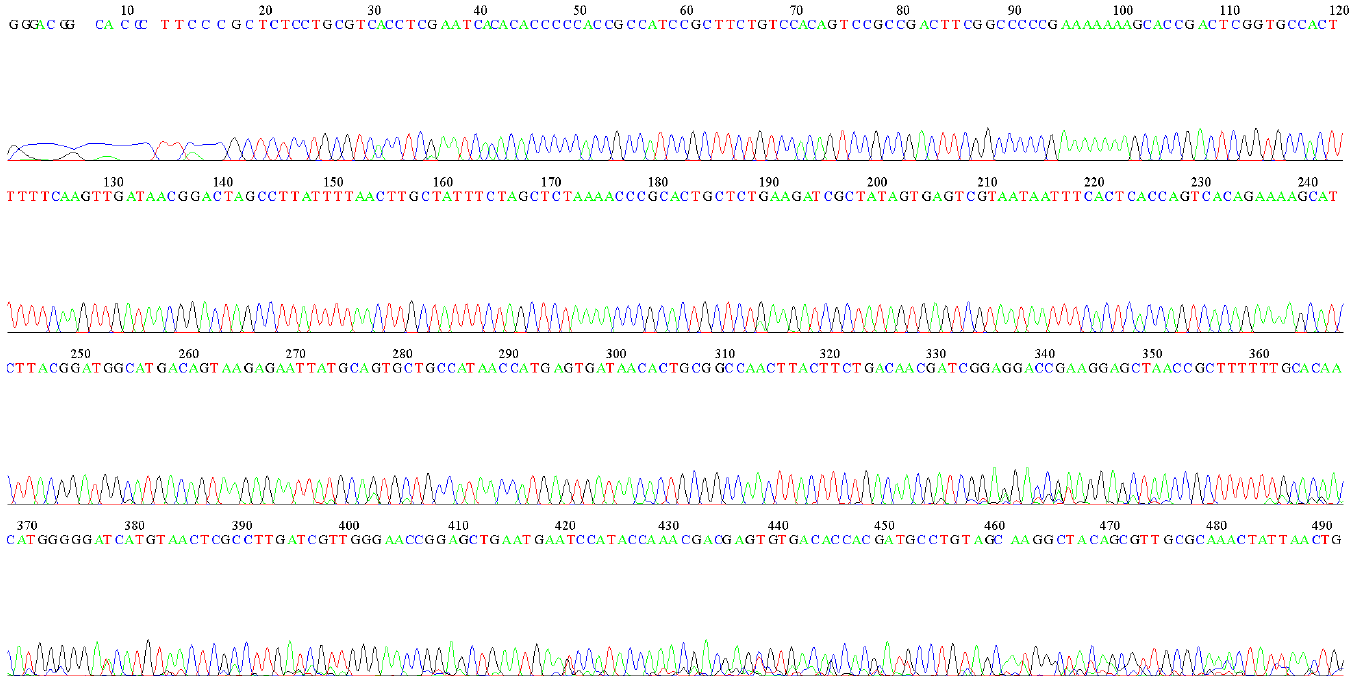


Primer_R


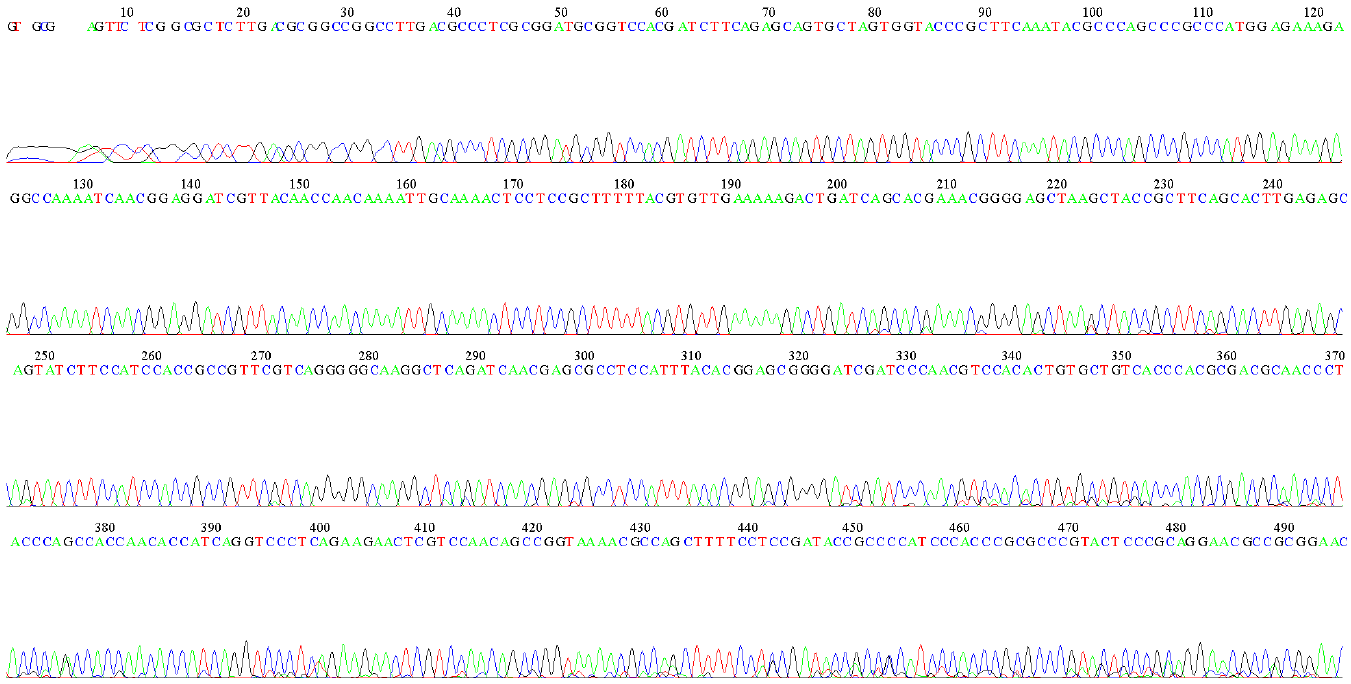


ΔCrFTSY_mV mutant 3

Primer_F


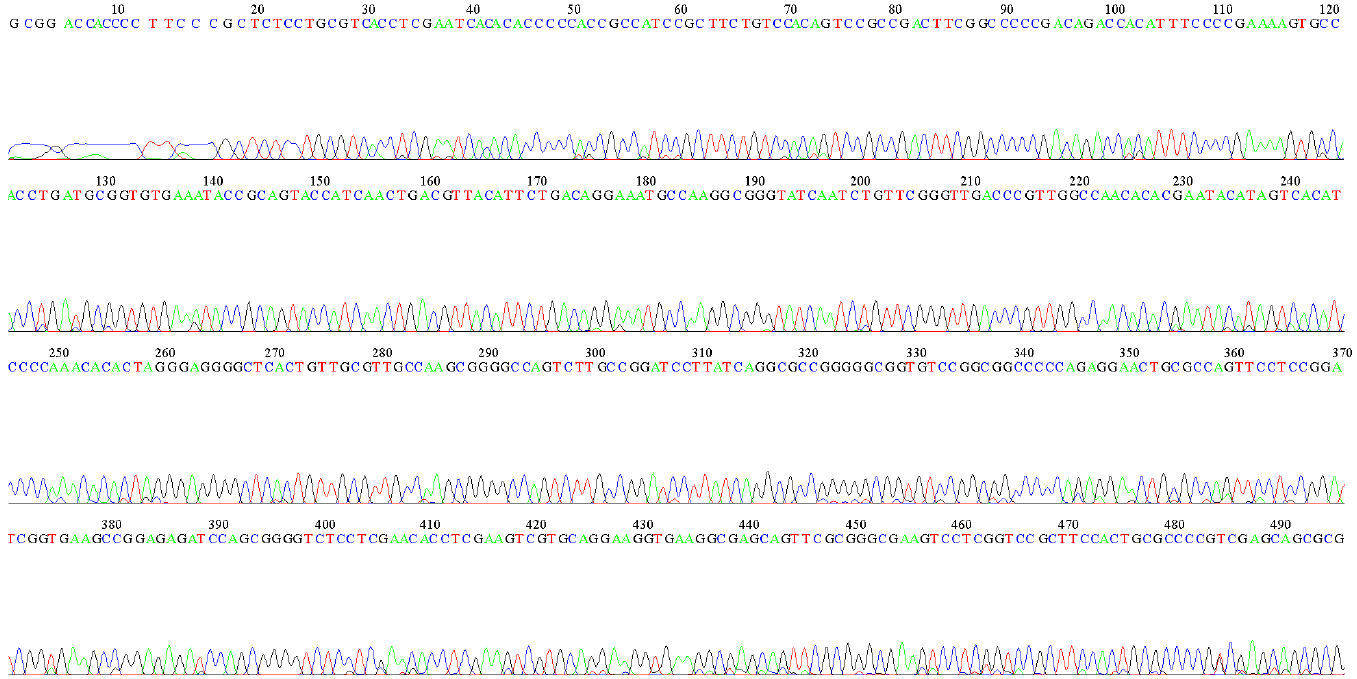


Primer_R


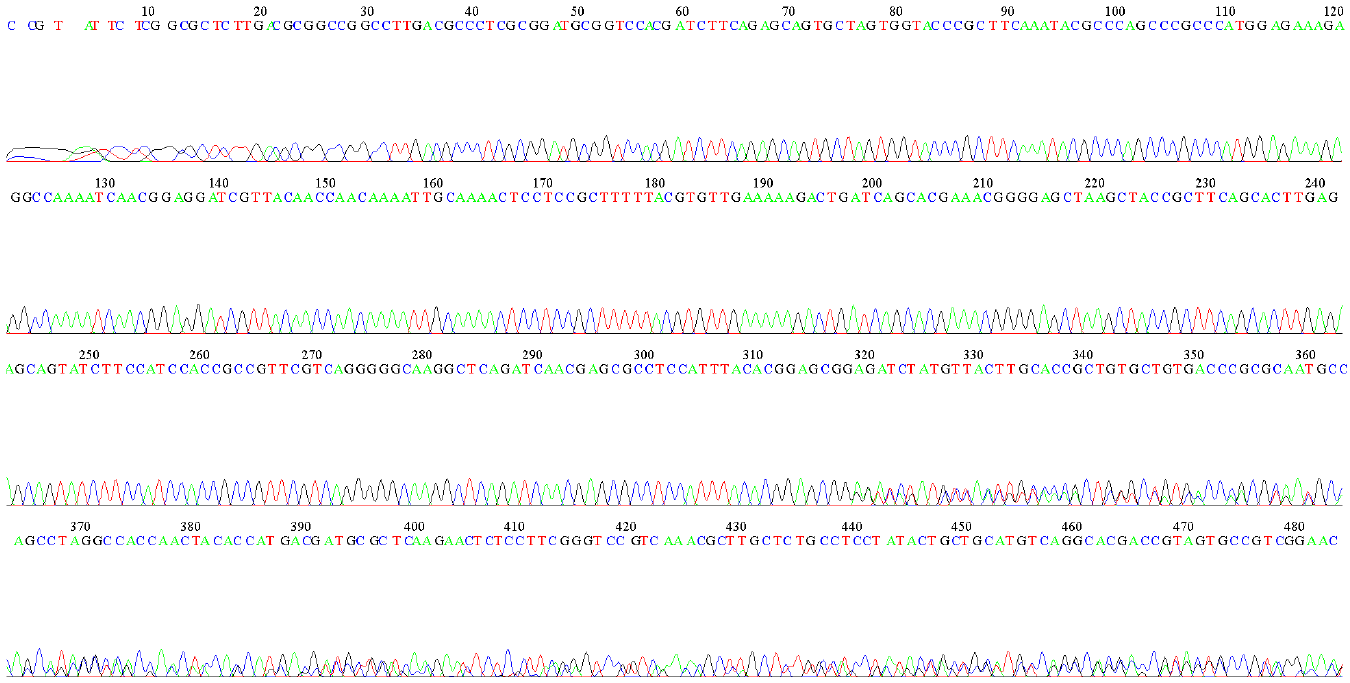


ΔCrFTSY_mV mutant 4

Primer_F


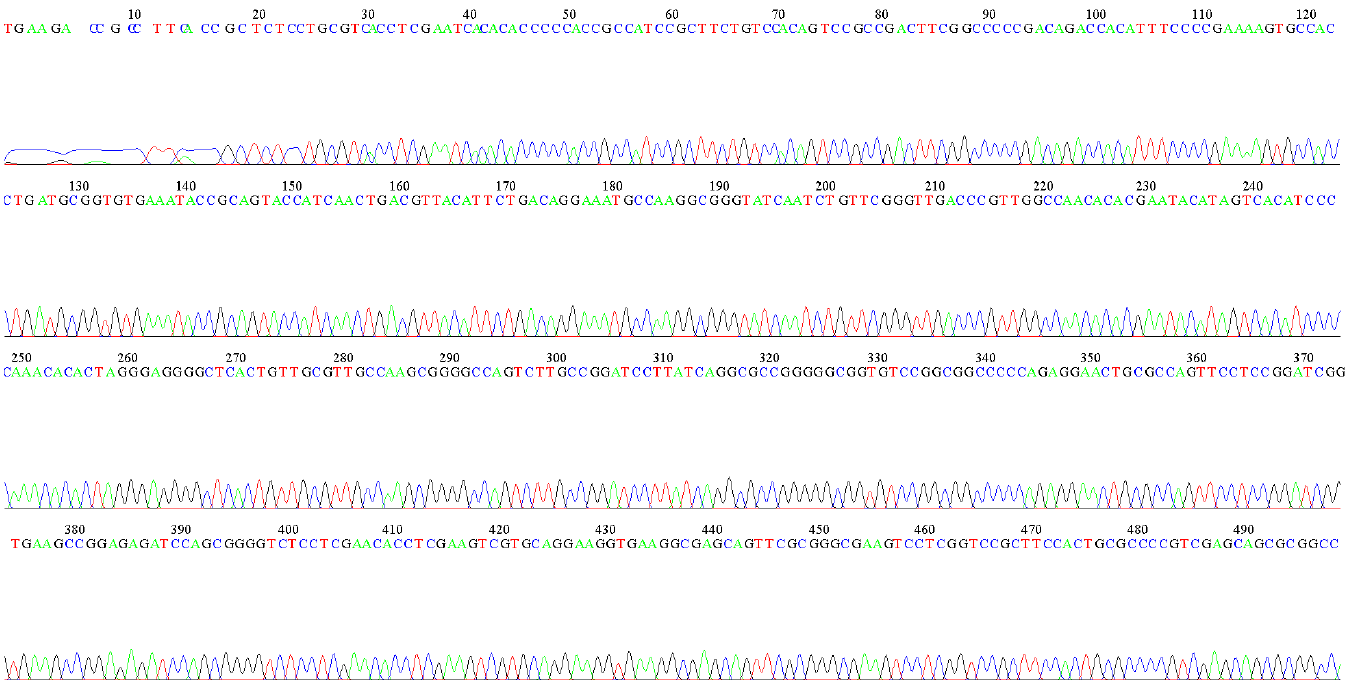


Primer_R


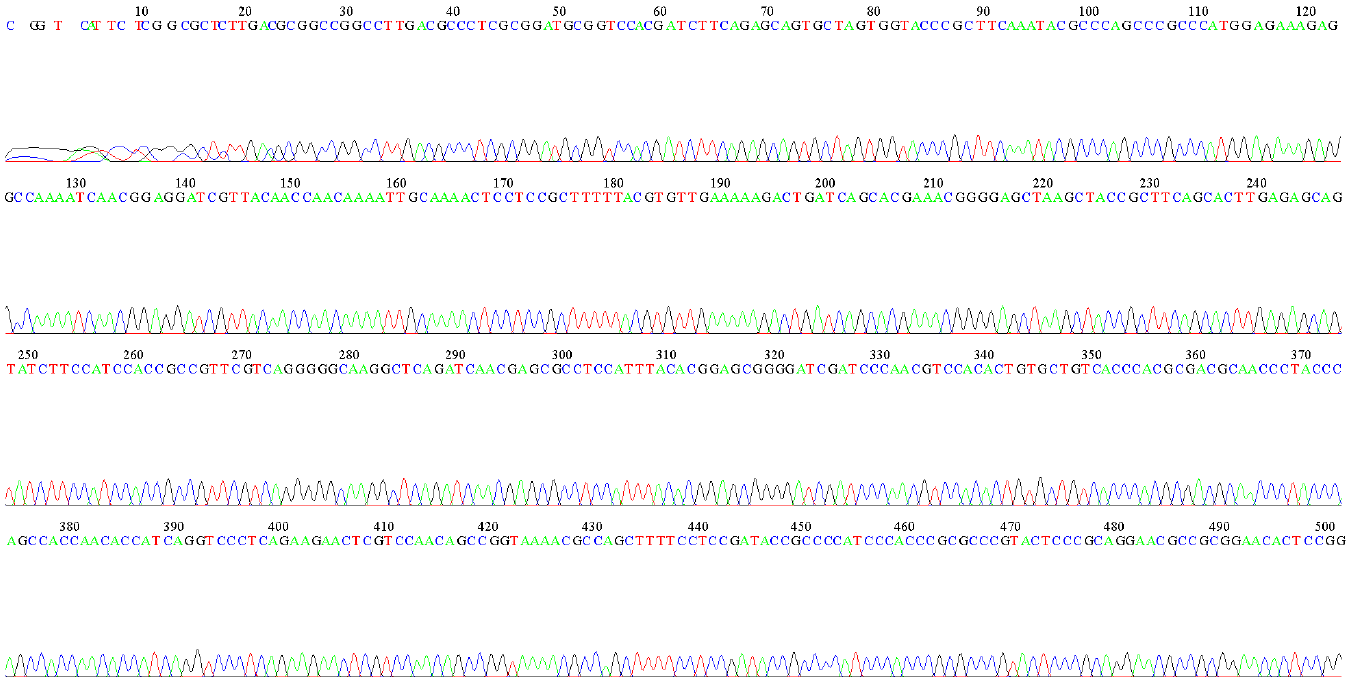

Supplement: Supplementary file 2 [file Data_Sheet_2.docx]
